# Biophysical analysis of *Pseudomonas*-phage PaP3 small terminase suggests a mechanism for sequence-specific DNA-binding by lateral interdigitation

**DOI:** 10.1093/nar/gkaa866

**Published:** 2020-10-30

**Authors:** Marzia Niazi, Tyler J Florio, Ruoyu Yang, Ravi K Lokareddy, Nicholas A Swanson, Richard E Gillilan, Gino Cingolani

**Affiliations:** Department of Biochemistry and Molecular Biology, Thomas Jefferson University, 1020 Locust Street, Philadelphia, PA 19107, USA; Department of Biochemistry and Molecular Biology, Thomas Jefferson University, 1020 Locust Street, Philadelphia, PA 19107, USA; Department of Biochemistry and Molecular Biology, Thomas Jefferson University, 1020 Locust Street, Philadelphia, PA 19107, USA; Department of Biochemistry and Molecular Biology, Thomas Jefferson University, 1020 Locust Street, Philadelphia, PA 19107, USA; Department of Biochemistry and Molecular Biology, Thomas Jefferson University, 1020 Locust Street, Philadelphia, PA 19107, USA; Macromolecular Diffraction Facility, Cornell High Energy Synchrotron Source (MacCHESS), Cornell University, 161 Synchrotron Drive, Ithaca, NY 14853, USA; Department of Biochemistry and Molecular Biology, Thomas Jefferson University, 1020 Locust Street, Philadelphia, PA 19107, USA

## Abstract

The genome packaging motor of tailed bacteriophages and herpesviruses is a powerful nanomachine built by several copies of a large (TerL) and a small (TerS) terminase subunit. The motor assembles transiently at the portal vertex of an empty precursor capsid (or procapsid) to power genome encapsidation. Terminase subunits have been studied in-depth, especially in classical bacteriophages that infect *Escherichia coli* or *Salmonella*, yet, less is known about the packaging motor of *Pseudomonas*-phages that have increasing biomedical relevance. Here, we investigated the small terminase subunit from three *Podoviridae* phages that infect *Pseudomonas aeruginosa*. We found TerS is polymorphic in solution but assembles into a nonamer in its high-affinity heparin-binding conformation. The atomic structure of *Pseudomonas* phage PaP3 TerS, the first complete structure for a TerS from a *cos* phage, reveals nine helix-turn-helix (HTH) motifs asymmetrically arranged around a β-stranded channel, too narrow to accommodate DNA. PaP3 TerS binds DNA in a sequence-specific manner *in vitro*. X-ray scattering and molecular modeling suggest TerS adopts an open conformation in solution, characterized by dynamic HTHs that move around an oligomerization core, generating discrete binding crevices for DNA. We propose a model for sequence-specific recognition of packaging initiation sites by lateral interdigitation of DNA.

## INTRODUCTION

The genome-packaging motor of tailed bacteriophages and herpesviruses is a multi-subunit nanomachine that catalyzes adenosine triphosphate (ATP)-dependent translocation of double-stranded DNA (dsDNA) inside an empty precursor capsid (also known as ‘procapsid’) (1–4). The packaging motor is formed by several copies of two non-structural proteins, TerL and TerS, which assemble onto a unique vertex of procapsid occupied by the dodecameric portal protein. At this vertex, the portal protein replaces a single penton, forming a channel for the passage of DNA, as well as a sensor for genome-packaging (5–7) and an anchoring site for the terminase complex. In certain phages, small nuclease-associated proteins called HNH-proteins facilitate the packaging reaction, possibly by interacting with TerL (8). Terminase subunits play a vital role in the life cycle of bacteriophages and herpesviruses. The packaging motor is, in fact, one of the most powerful motors in nature, responsible for active DNA-packaging at a rate that exceeds ∼2000 bp/s in phage T4 (9). TerL is a bifunctional enzyme containing an N-terminal DNA-translocating ATPase domain and a C-terminal nuclease domain responsible for cleaving the viral genome (2). TerL is always monomeric in solution (10–13) but assembles into a pentamer upon binding to the procapsid of T4 (14), T7 (15) and phi29 (16,17), generating a symmetry mismatch with the portal vertex (18,19). In contrast, TerS is a DNA recognition subunit that binds packaging initiation sites (referred to as *pac* or *cos*) in preparation for genome packaging (20). TerS also stimulates the ATPase activity of TerL (21–23), while repressing the large terminase nuclease activity (24,25).

Despite decades of research, the mechanisms of TerS binding to DNA and its role in motor initiation are not fully understood. The substrate for genome packaging is a concatemer DNA molecule that consists of multiple genome units covalently linked together. Terminase subunits use different packaging strategies to process concatemeric DNA and insert a single genome inside a procapsid. A major difference exists between phages that use a *cos* versus a *pac* sequence (26). For *cos* packagers, exemplified by phage λ (20), the *cos* site is the point of junction between two genomes in a concatemer of phage DNA. Each genome has cohesive ends: phage λ carries 12 base-long single-strand extensions surrounding its chromosome that anneal upon entry into a host cell. The DNA segment containing the DNA packaging signals and the annealed cohesive ends is called *cos*. These *cos* phages produce virion DNAs through TerL’s introduction of precisely staggered nicks in the *cos* sequence, which serves both as the packaging initiation site and a specific packaging termination sequence. As a result, *cos* packagers package accurately one genome unit at a time, without terminal duplications. In contrast, the *pac* sequence is found in viruses that use the head-full packaging mechanism, among which P22 is perhaps the best-characterized example (27). In these phages, which typically also result in generalized transduction, the *pac* site is the recognition site for TerS. The packaging reaction is initiated by a first cut in the proximity of a *pac* site that consists of a 22-bp asymmetric sequence in the TerS gene for P22 (27), or multiple points of contact flanking the site where TerL makes an initial cut in SPP1 (28). Genome packaging proceeds possessively in *pac* packagers and is terminated by a non-specific cut when the procapsid is full (hence the name ‘head-full’ packaging). The termination cut is also the start of the packaging for the next chromosome along the concatemer, which results in viral chromosomes that have a terminal redundancy and individual chromosomes that are circular permutations of the unique viral sequence. Also, in SPP1, the *pac* site is estimated to be used only once every four packaging events (29).

The interaction of TerS with *pac* or *cos* sites varies from phage to phage (26), and TerS specificity is not always recapitulated *in vitro*, especially in *pac* packagers like P22 (30,31), Sf6 (32,33), P76–26 (25), SF6 (34) and SPP1 (35). These phages typically lack terminase sequence-specificity, and their TerSs usually associate weakly with DNA *invitro*. However, a previous report on SPP1 TerS found this protein binds DNA with nanomolar affinity and induces significant bending in the double helix (28). This effect was inhibited by distamycin, a minor groove binder that causes local distortion of the minor groove of DNA (36). These findings led to a model whereby SPP1 TerS recognizes the bent structure of DNA rather than the *pac* DNA sequence. Instead, the TerS from phage λ, a *cos* packager (37–39), binds specifically to its cognate *cos* sequence *in vitro*. The different affinity of TerS for DNA between *pac* and *cos* packagers reflects the different packaging strategy and the fact that *cos* sequence serves as both the packaging initiation site and a specific packaging termination sequence. In T4, TerS is dispensable for packaging in a defined packaging assay carried out with non-physiological concentrations of purified T4 components (40,41). Also, the physical association of terminase subunits, and whether TerL and TerS remain assembled into a complex during genome-packaging, is poorly understood. In many phages, the two subunits fail to interact stably in solution, with two noticeable exceptions. In phage λ, TerS (gpNu1) forms a hetero-trimer bound to a monomer of TerL (gpA1), that further assemble into tetramers (42). In P22, the terminase complex can be isolated from *Salmonella* infected cells (43) or formed in *Escherichia coli* (10) and purified to homogeneity. Thus, although the general architecture of terminase subunits is conserved in the virosphere, the specific mechanisms by which TerS and TerL interact to promote genome-packaging may have diverged significantly in different phages.

*Pseudomonas aeruginosa* phages 1–3 (PaP1, PaP2 and PaP3) and the close relative phage NV1 were isolated from hospital sewage (44,45). PaP2 and PaP3 are temperate phages while PaP1 is virulent. NV1 is very similar to the intron-containing lytic *Pseudomonas* phage LUZ24, also isolated from hospital sewage, that shares 71% sequence similarity to phage PaP3 (46). The complete sequence of PaP3, NV1 and LUZ24 genomes (∼45.5 kbs) confirmed similarity to classical *Podoviridae*, like P22 or T7 (47). These phages are built by an icosahedral capsid made of a coat and scaffolding protein and encode components of a short ∼12 nm tail, including a portal protein and tail spikes. PaP3 TerL, previously named p03, shares the classical domain signature of large viral terminases, with an N-terminal ATPase and a C-terminal nuclease domain (48). Instead, PaP3 TerS (p01) has limited sequence similarity with TerSs from *E. coli* or *Salmonella* phages but is ∼22% identical to the *Bacillus*-phage SF6 TerS. Purified PaP3 TerS retains the two primary activities of small terminases: it binds the *cos* packaging initiation site and stimulates the ATPase activity of TerL (48).

In this paper, we present the first three-dimensional structure of a *Pseudomonas*-phage TerS that we studied using hybrid structural methods. We demonstrate PaP3 TerS binds DNA through a highly dynamic N-terminal helix-turn-helix (HTH)-motif that adopts a different conformation in solution versus in crystal. We present a model for sequence-specific DNA recognition by lateral interdigitation of DNA.

## MATERIALS AND METHODS

### DNA and plasmids

Synthetic genes encoding PaP3 TerS (Gene ID: 2700603, or *orf1*), NV1 TerS (Gene ID: 40099729) and LUZ24 TerS (Gene ID: 5896731) were purchased from Genewiz and ligated between BamHI and XhoI restriction sites of the expression vector pGEX-6P (GE Healthcare) (plasmid pGEX-6P-TerS). PaP3 TerL (Gene ID: 2700601, or *orf3*), also synthesized by Genewiz, was ligated in a pET28a (Novagen) expression vector between BamHI and XhoI (plasmid pET28a_PaP3-TerL). PaP3 ΔC122-TerS was constructed by introducing a stop codon at position E122 of TerS (plasmid pGEX-6P-ΔCTerS). Ala mutants DM-TerS (K17/K19), TM-TerS (K17/K19/K33) and pAla-TerS (K17/K19/K33/R49/R56/K57) were generated using site-directed mutagenesis. All plasmids were sequenced to confirm the fidelity of the DNA sequence. Eurofins Genomics LLC synthesized DNA fragments corresponding to the PaP3 cohesive (*cos*) site (5′-GCCGGCCCCTTTCCGCGTTA-3′) and complementary fragment, both 5′ Cy3-labeled. The single-stranded complementary *cos* oligos were annealed to generate double-stranded DNA (dsDNA). A non-specific 5′ Cy3-labeled 24-bp dsDNA (5′-GCACTGCAGTAACTTGTCAGTCAT-3′) generated from single-stranded oligos was used as a negative control.

### Biochemical techniques

Expression plasmids for all TerSs were transformed and expressed in LOBSTR-BL21 (DE3) *E.coli* strain in the presence of ampicillin. Bacterial cultures were grown in LB medium at 37°C until A_600_ = ∼0.3 when the temperature was reduced to 18°C. The cultures at A_600_ = 0.6 were induced with 0.5 mM IPTG for 12–16 h. Cell pellets expressing TerS were sonicated in Lysis Buffer (20 mM Tris–HCl, pH 8.0, 250 mM NaCl, 2.5% (v/v) glycerol, 3 mM β-Mercaptoethanol, 1 mM phenylmethylsulfonyl fluoride (PMSF)). TerS was purified by affinity chromatography on Glutathione Resin (GenScript) and incubated overnight with 100 U of PreScission Protease in PP buffer (20 mM Tris–HCl, pH 7.0, 150 mM NaCl, 1 mM dithiothreitol (DTT), 1 mM ethylenediaminetetraacetic acid (EDTA), 0.1% (v/v) TWEEN^®^ 20). Cleaved, untagged TerSs were recovered, diluted in Buffer A (20 mM Tris–HCl, pH 8.0, 2.5% (v/v) Glycerol, 3 mM β-Mercaptoethanol, 0.1 mM PMSF) and passed on a 1 ml HiTrap™ Heparin HP Column (GE Healthcare) where the protein was eluted with a linear gradient from 0–100% Buffer B (20 mM Tris–HCl, pH 8.0, 1 M NaCl, 2.5% (v/v) Glycerol, 3 mM β-Mercaptoethanol, 0.1 mM PMSF). TerS eluted as two peaks at 550 mM (peak 1) and 850 mM (peak 2) NaCl. Fractions from either peak were pooled, concentrated with 30 kDa Millipore concentrators, and injected on a Superose 6 column (GE Healthcare) pre-equilibrated with GF buffer (20 mM Tris–HCl pH 7.5, 150 mM NaCl, 5 mM β-Mercaptoethanol, 2.5% (v/v) glycerol). pGEX-6P-ΔCTerS and all point mutants in the HTH were expressed and purified as described for the full-length PaP3 TerS. Heparin-chromatography was omitted during the purification of Ala-mutants because of the decreased binding of these mutants to DNA. PaP3 TerL (M.W. ∼56.7 kDa) cloned in a pET28a vector was expressed in LOBSTR-BL21 (DE3) *E. coli* in the presence of Kanamycin. TerL was purified by metal affinity chromatography on Ni-beads (Genscript). TerL elutions were injected directly on a Superose 200 16/60 column (GE Healthcare) pre-equilibrated with GF buffer. Eluted fractions containing TerL were concentrated using a 30 kDa Millipore concentrator.

### Electrophoretic mobility shift assay (EMSA)

Electrophoretic mobility shift assay (EMSA) was carried out on a 1% native agarose gel, or a 4–16% acrylamide gel (Novex™ TBE Gels). PaP3 TerS from peak 2 was used in both assays. WT-TerS or the various mutants were mixed with Cy3-*cos* or Cy3-*scr* oligonucleotides and incubated at 37°C for 30 min. The protein:DNA mixture was resolved either on a 1% agarose gel (49) or a 4–16% acrylamide gel (Novex™ TBE Gels) in the presence of 0.5× TBE buffer (45 mM Tris base, 45 mM Boric Acid, 1 mM EDTA) at 4°C. The Cy3 signals were measured using a ChemiDoc MP (Bio-Rad) at 602 nm. The intensity of the bands was quantified using ImageJ (49). The fraction of TerS bound to DNA was calculated by dividing the total intensity of all bands in each lane by that of the bands representing protein–DNA complexes. The plot was generated using GraphPad Prism 8 based on three independent experiments.

### Crystallographic methods

TerS from peak 1 and 2 were concentrated to 7.5 mg ml^−1^ and crystallized using the vapor diffusion hanging drop method by mixing 2 μl of purified protein with an equal volume of crystallization solution. Only peak 2 yielded diffracting crystals. PaP3 TerS crystallized in the presence of 100 mM KCl, 25 mM MgCl_2_, 50 mM Na-Cacodylate trihydrate pH 6.0, 15% (v/v) 2-propanol. NV1 crystals were obtained in the presence of 0.1 M Succinic acid pH 7.0, 15% w/v Polyethylene glycol 3350. Crystals were harvested in nylon cryo-loops, cryo-protected with 27% ethylene glycol and flash-frozen in liquid nitrogen. Complete diffraction data were collected at beamline 9–2, at Stanford Synchrotron Radiation Lightsource (SSRL) for PaP3 and Advanced Photon Source (APS) beamline 23-ID-D for NV1, both on a Dectris Pilatus 6M detector (Table 2). PaP3 TerS structure was solved by molecular replacement (MR) using the oligomerization core of phage SF6 (PDB ID: 3ZQP) as a search model, as implemented in PHASER (50). A partial model that accounts for less than half of all residues in PaP3 TerS was built using *phenix.autobuild* (51) and completed manually using Coot (52). The structure was then subjected to additional cycles of positional and isotropic B-factor refinement using *phenix.refine* (51) enforcing 9-fold non-crystallographic symmetry (NCS) torsional restraints and alternating cycles of refinement with Refmac5 using jelly-body restraints (53). When the model reached an *R*_free_ ∼30%, the electron density was sharpened and the model further subjected to real-space refinement using phenix.real_space_refine (54), followed by additional positional refinement using *phenix.refine*. The final model has an *R*_work_/*R*_free_ of ∼25.06/27.49, at 3.0 Å resolution, and includes residues 13–121. The model has excellent geometry (Table 2), with 100% residues in the most favored regions of the Ramachandran plot and root means square deviation (RMSD) of bond lengths and angles of 0.005 Å and 0.953°, respectively. The overall B-factor of the refined model is high, especially at the N-termini (B∼125 Å^2^), consistent with the structural plasticity of the HTH-motifs that adopt asymmetric conformations. NV1 TerS was phased by MR using PaP3 TerS as a phasing model using PHASER (50). NV1 diffraction data are weak and suffer from pseudo-translation and twinning, as detected by *phenix.xtriage* (51), which prompted us to solve the molecular replacement problem in space group P1. PHASER readily identified nine copies of the nonameric TerS arranged as a superhelix (Supplementary Figure S5). The AU content was then subjected to reciprocal and B-factor refinement in Refmac5 (53) and then real-space refinement in Phenix (51) enforcing 81-fold NCS torsion restraints. The final model has an *R*_work_/*R*_free_ of 25.9/29.1% using all data between 10–3.95 Å resolution. Final stereochemistry was validated using MolProbity (55) (Table 2). All ribbon models were generated using PyMol (56). Structural homologs of PaP3 TerS were identified using the DALI server (57).

### Negative stain electron microscopy

A total of 5 μl of LUZ24 TerS from peak 2 at 0.025 mg ml^−1^ were adsorbed for 1 min to carbon-coated 400-mesh copper grids (CF400-CU, EMS) glow-discharged for 2 min at 25 mA using an easiGlow (PELCO). The grids were quickly washed with three 25 μl MilliQ water droplets, followed by staining with 25 μl 2% uranyl acetate for 10 s and again for 1 min. Grids were blotted with filter paper and air-dried for at least 5 min before screening. Images were collected on a FEI Tecnai T12 electron microscope at the Electron Microscopy Resource Lab (EMRL) at the Perelman School of Medicine, University of Pennsylvania. The microscope was operated at 100 kV, at 67 000× magnification with a pixel size of 1.66 Å and defocus range of −0.5 to −1.5 μm. Particle picking, steps of classification, initial model generation and 3D refinement were performed using RELION-3.1 (58). LUZ24 TerS final 3D refinement was performed with C9 symmetry imposed.

### Analytical ultracentrifugation sedimentation velocity

AUC-SV analysis was carried out using a Beckman XL-A Analytical Ultracentrifuge. TerS peak 1 and peak 2 at ∼75  μM (corresponding to ∼1.25 mg ml^−1^) were dissolved in AUC buffer (20 mM Tris–HCl pH 7.5, 150 mM NaCl, 3 mM DTT, 2.5% (v/v) glycerol) and spun at 40 000 rpm at 6 °C. Absorbance values between 280 nm were fit to a continuous sedimentation coefficient (c(s)) distribution model in SEDFIT (59). Data were visualized and presented using GUSSI (University of Texas Southwestern Medical Center).

### Size exclusion chromatography coupled to small angle X-ray scattering

Size exclusion chromatography coupled to small angle X-ray scattering (SEC-SAXS) analysis was performed at ID7A1 station at MacCHESS, which is equipped with an AKTA Pure FPLC system (GE Healthcare). The PaP3 TerS sample and NV1 TerS samples from peak 2 were loaded at 1.7 and 2.0 mg ml^−1^, respectively, on a Superdex 200 10/300 GL column (GE Healthcare) equilibrated in 20 mM Tris–HCl pH 8.0, 150 mM NaCl, 2.5% glycerol and 0.5 mM TCEP. SAXS data were recorded on an EIGER 4M detector (Dectris Ltd. Baden, Switzerland) *in vacuo* at 0.5 s per frame with a fixed camera length of 1.718 m and 10.04 keV (1.234 Å) energy allowing the collection of the angular range q between 0.008 and 0.54 Å^−1^. Primary reduction of the SAXS data was performed using RAW (60), and ATSAS software (61). To minimize the effects of damaged material accumulating on the X-ray sample window and to help compensate for any baseline drift, the buffer profile was constructed by averaging PaP3 and NV1 frames before (495–514 and 280–299, respectively) the sample peaks (538–573 and 515–550, respectively). The Guinier plots of the subtracted profiles were linear to the lowest measured q value (62). GNOM (63) was used to calculate *P(r)* plots from the scattering data. The PaP3 and NV1 maximum diameter (*D*_max_) value of 131 Å was chosen so that the *P(r)* function fell gradually to zero at *r* = *D*_max_. *Ab initio* model calculations to generate an average electron density from solution scattering data were done using DENSS (64), as implemented in RAW (60). The DENSS density was improved by applying rotational symmetry (Supplementary Table S1). For both PaP3 and NV1-TerS, 9-fold symmetry gave the best FSC resolution. Docking of PDB models inside the SAXS density was done manually and improved by rigid-body refinement using Chimera (65) and *phenix.real_space_refine* (66). Theoretical solution scattering curves were calculated using the FoXS web server (67). The χ^2^ between TerS SAXS electron density and the atomic models is *χ*^2^ = 2.33 for PaP3 and *χ*^2^ = 2.55 for NV1. The NV1 crystal structure was manually modeled in PyMoL by rigid-body rotating the helical hairpin α1–α2 by 60° around helix α3 (see ‘open’ model in Figure 5C). The ‘open’ model was then manually docked within the SAXS density, and rigid-body refined in Chimera (‘fit-into-volume’ command) (65) where the HTH matched the outer lobes of the electron density with an improved *χ*^2^ = 1.04 (Supplementary Figure S8). SEC-SAXS data collection and analysis statistics are in Table 3. Non-linear Poisson-Boltzmann electrostatic calculations were performed using APBS-PDB2PQR tools (68).

## RESULTS

### Sedimentation analysis of TerSs from *Pseudomonas* phages PaP3, NV1 and LUZ24

**Figure 1.**
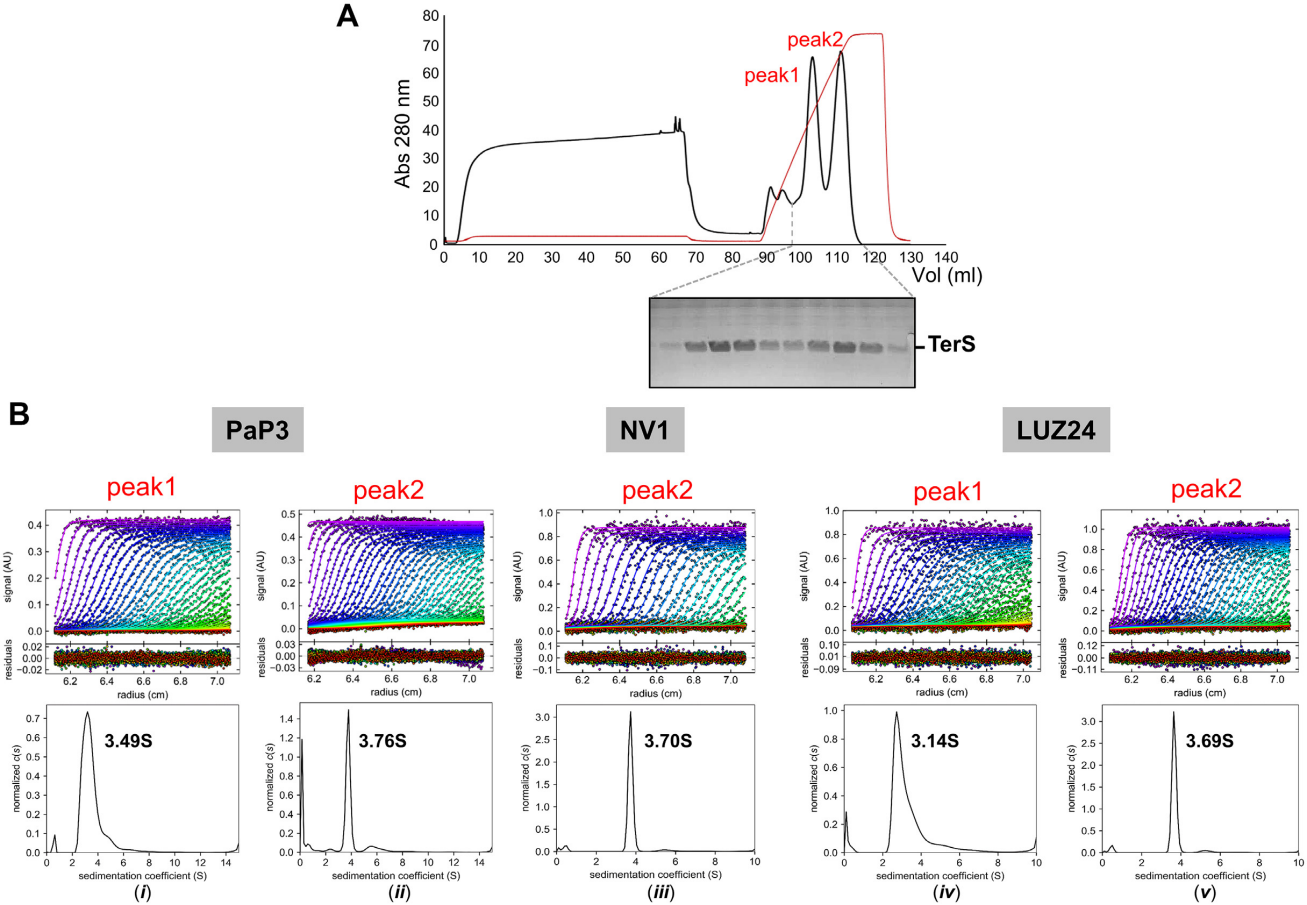
AUC sedimentation analysis of three *Pseudomonas*-phage TerSs. (**A**) Purification of PaP3 TerS by heparin chromatography yielded two major species in peak 1 and peak 2 that eluted at 550 and 850 mM NaCl, respectively. The two species were indistinguishable by SDS-PAGE. (**B**) AUC-SV profiles of TerS from peak 1 or peak 2 for PaP3 (**i** and **ii**), NV1 (**iii**) and LUZ24 (**iv** and **v**). Top panel: raw absorbance at 280 nm plotted as a function of the radial position. Middle panel: the residuals between the fitted curve and raw data. Bottom panel: the fitted distribution of the apparent sedimentation coefficient (S).

TerS from three *Podoviridae* phages that infect *P. aeruginosa*, namely, PaP3 (47), NV1 (45) and LUZ24 (46) have high sequence identity, ranging between 65.1% (PaP3 and NV1) and 89.5% (PaP3 and LUZ24) and nearly identical amino acid coverage (∼155 residues) (Supplementary Figure S1). We expressed all three TerSs in bacteria and purified the relative recombinant proteins from soluble lysates. To enrich for the DNA-binding conformation of TerS, we passed partially purified TerSs over a heparin affinity resin, which mimics DNA. This purification step identified three populations of TerS: one that bound heparin with low affinity but had a low Abs_280/260_ ratio indicative of a nucleic acid-contamination, and two high-affinity binding species eluted with ∼550 mM (‘peak 1’) and ∼850 mM (‘peak 2’) sodium chloride. Peak 1 and peak 2 had a roughly equal quantity of protein for PaP3 and LUZ24 (Figure 1A and Supplementary Figure S2B), whereas most of NV1 TerS eluted in peak 2 (Supplementary Figure S2A). Furthermore, the two populations of TerS had identical electrophoretic mobility on gel, ruling out peak 1 is a degradation product of peak 2.

To assess the molecular weight and oligomerization of TerS, we subjected TerS eluted in peak 1 and 2 to analytical ultracentrifugation sedimentation velocity analysis (AUC-SV). All TerSs recovered in peak 2 sedimented as ∼3.7 S species (Figure 1B (ii), (iii) and (v)) equivalent to ∼155 kDa, which is consistent with a nonameric assembly of a ∼17 kDa subunit (Table 1). In contrast, TerS from peak 1 had a broader sedimentation profile and a slightly smaller sedimentation coefficient: 3.49 S for PaP3 TerS and 3.14 S for LUZ24 (Figure 1B (i) and (iv)). TerS in peak 1 and 2 were indistinguishable by sodium dodecyl sulphate-polyacrylamide gel electrophoresis (SDS-PAGE) analysis, but the species in peak 1 was smaller than peak 2 and more heterogeneous in solution (Table 1). Thus, three related small terminase subunits from *Pseudomonas* phages PaP3, NV1 and LUZ24 are polymorphic in solution but adopt a nonameric quaternary structure in the high affinity heparin-binding conformation.

**Table 1.** Biophysical parameters measured using AUC-SV

	PaP3 TerS		NV1 TerS		LUZ24 TerS	
AUC-SV	Peak 1	Peak 2	Peak 1	Peak 2	Peak 1	Peak 2
Protein concentration (mg ml^−1^)	1.2	1.3	n.a.	2.2	2.3	2
*Apparent* sedimentation coef., s (S)	3.49 ± 0.20	3.78 ± 0.02	n.a.	3.68 ± 0.03	3.14 ± 0.20	3.68 ± 0.03
*Absolute* sedimentation coef., s_20,w_ (S)	3.97	4.28	n.a.	4.21	3.58	4.20
Theoretical monomer M.W. (kDa)	17.01		17.73		17.01	
Estimated M.W. (kDa)	154.4 ± 2.57	152.20 ± 0.57	n.a.	164.04 ± 3.12	103.7 ± 3.50	156.84 ± 2.50
Possible oligomeric state (theoretical M.W., kDa)	*Heterogeneous*	9-mer (153.1)	n.a.	9-mer (159.6)	*Heterogeneous*	9-mer (153.0)
Frictional ratio, *f/f_0_*	2.55	2.35	n.a.	2.47	2.15	2.39
Abundance in each population (%)	95	61.2	n.a.	92.5	90.7	91.4

### Structural analysis of TerS from PaP3, NV1 and LUZ24

We attempted the crystallization of all TerS species isolated on heparin but obtained diffracting crystals only of PaP3 and NV1 TerS from peak 2. Despite the high sequence identity, the three proteins behaved very differently. PaP3 TerS crystals diffracted to ∼3 Å resolution with one nonamer in the asymmetric unit (AU) (Supplementary Figure S3 and Table 2). NV1 TerS crystallized in a large monoclinic unit cell with five nonamers in the AU that diffracted weakly to 3.95 Å resolution (Table 2). LUZ24 TerS yielded over a dozen crystal forms, none of which diffracted X-rays better than 15 Å.

**Table 2.** Crystallographic data collection and refinement statistics

	PaP3 TerS	NV1 TerS
**Data collection**		
Beamline	SSRL 9–2	APS 23-ID-D
Wavelength (Å)	0.976	1.033
Space group	P2_1_2_1_2_1_	P1
Cell dimensions		
*a*, *b*, *c* (Å)	108.3, 121.1, 129.6	119.2, 119.1, 382.9
*α*, *β*, *γ* (°)	90.0, 90.0, 90.0	89.8, 90.0, 119.9
Reflections (tot/unique)	2,643,598/32,945	1,114,004/150,584
Resolution (Å)	50.0–3.0 (3.11–3.10)	15.0–3.95 (4.13–3.95)
Completeness (%)	98.4 (99.6)	94.0 (93.0)
Redundancy	6.4 (6.4)	1.6 (1.5)
*R*_sym_	5.9 (88.2)	21.5 (65.2)
R_pim_	3.5 (69.6)	20.5 (51.2)
*I* / σ*_I_*	49.1 (1.7)	3.2 (1.2)
CC1/2	0.42	0.35
**Refinement**		
PDB ID	**6W7T**	**7JOQ**
Resolution limits (Å)	15.0–3.0	10.0–3.95
No. of reflections	26,062*	142,947
*R*_work_/*R*_free_^a^	25.1/27.5	25.9/29.1
No. of protein atoms	7422	62,617
No. chains	9	81
No. of solvent molecules	0	0
Ramachandran plot (%) core/allow/gen. allow/disallowed Rms deviations from ideal	95.9/4.1/0.0	93.3/6.7/0.0
Bond lengths (Å)	0.005	0.003
Bond angles (°)	0.953	0.775
MolProbity score	2.3	1.9
MolProbity clash score	15.7	8.6

Values in parentheses are for the highest-resolution shell.

^a^The *R*_free_ value was calculated using ∼2000 reflections selected in 20 thin resolution shells.

*|F_obs_|σ_Fobs_ > 3

**Figure 2.**
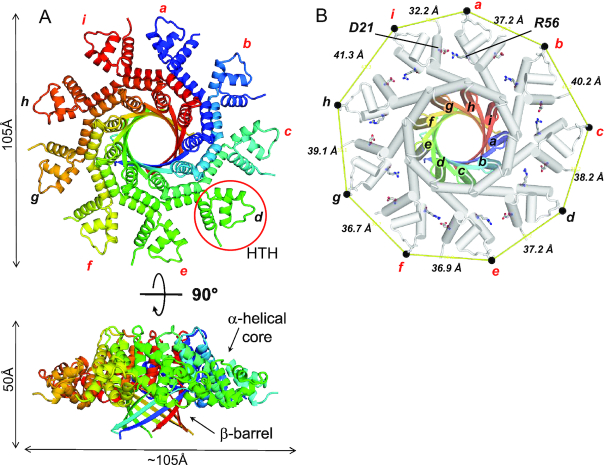
Crystal structure of PaP3 TerS. (**A**) Top and side views of the quaternary structure of PaP3 TerS. The overall diameter of the molecule is 105 Å, with a height of 50 Å. One of the nine HTH motifs is circled in red. (**B**) Top view of TerS nonamer with helices shown as cylinders. The oligomerization core is color-coded as in panel A and the rest of TerS in gray. The nine subunits are labeled ‘*a’* through ‘*i*’. A black dot indicates the position of N45, and continuous yellow lines represent the distance between protomers. Only six TerS chains (colored in red) make a salt bridge between R56 and D21.

We phased PaP3 TerS diffraction intensities using the oligomerization core of SF6 TerS (34), a distant small terminase from a *Bacillus* phage that is only ∼22% identical in amino acid sequence and about half of the size of PaP3 TerS (Supplementary Figure S4A). An initial molecular replacement (MR) solution was gradually improved and entirely rebuilt, yielding a complete atomic model of PaP3 TerS currently refined to an *R*_work_/*R*_free_ of 25.0/27.4%, at 3.0 Å resolution (Figure 2A, Supplementary Figure S4B and Table 2). The RMSD between the search model and the final refined model is >5Å (Supplementary Figure S4A and C), underscoring the power of MR in phasing diffraction intensities from low-homology and partial atomic models. The structure of NV1 TerS was then solved by MR using the model of PaP3 TerS and refined to an *R*_work_/*R*_free_ of 25.9/29.1% at 3.95 Å resolution, enforcing 81-fold NCS torsion restraints (Supplementary Figure S5A and Table 2). NV1 and PaP3 TerSs are virtually identical (RMSD ∼ 1.1) (Supplementary Figure S5A), as expected for two proteins that are ∼65% identical in amino acid sequence. We also determined a low-resolution reconstruction of LUZ24 TerS using single particle analysis of negatively stained micrographs, which revealed a similar fold (Supplementary Figure S6). For a detailed description of the first *Pseudomonas* phage TerS, we will focus on PaP3 TerS, which we refined at 3.0 Å resolution.

### The 3D-structure of PaP3 TerS reveals profound structural asymmetry

PaP3 TerS folds into a hollow nonameric ring of mixed α/β structure that resembles a mushroom (Figure 2A). The quaternary structure of TerS consists of an apical gear-like ring ∼105 Å in diameter sitting onto a 9-stranded β-barrel ∼50 Å in height, similar to that found in β-porins (69). A search for structural homologs using DALI (57) identified a new putative nonameric TerS from a prophage of *Bacillus cereus* (PDB ID: 2AO9, *Z*-score 5.8), followed by TerSs from the *Bacillus* phage SF6 (PDB ID: 3ZQP, *Z*-score 5.5), which we used as a search model for MR, and the thermophilic phage G20c (PDB IDs: 6EJQ and 4XVN, *Z*-score 3.3). PaP3 TerS has a total solvent-accessible surface area of 51,050 Å^2^, almost identical to P22 TerS (∼51,200 Å^2^) (31,70), which does not appear among the top ten most similar structural homologs (*Z*-score ∼2.1). Unlike P22, PaP3 TerS lacks a ‘dome-domain’ (Supplementary Figure S7) and is pronouncedly asymmetric (Figure 2B). The RMSD between subunits varies between 1.23 Å for chains *a-h*, the most similar and 1.67 Å, for chains *a*-*i*, the most dissimilar. This asymmetry is generated by an uneven pattern of lateral contacts between neighboring protomers. Three pairs of contacting subunits (labeled as *i:a*, *b:c* and *e-f* in Figure 2B) make a close-distance (e.g. 2.5–3.5 Å) salt bridge between R56 and D21 projecting from juxtaposed protomers. In contrast, the same two residues are too far for bonding in the other subunits (e.g. between 4.6 and 14.4 Å) (Figure 2B). The global asymmetry of PaP3 TerS can be described by the uneven distance between equivalent residues projecting at the perimeter of the oligomer (Figure 2B). For instance, the distance between the side-chain nitrogen atom of N45 between neighboring protomers varies between 32.2 Å and 41.3 Å for subunits *i:a* and *h:i*, respectively (Figure 2B), underscoring the profound asymmetry of this assembly.

**Figure 3.**
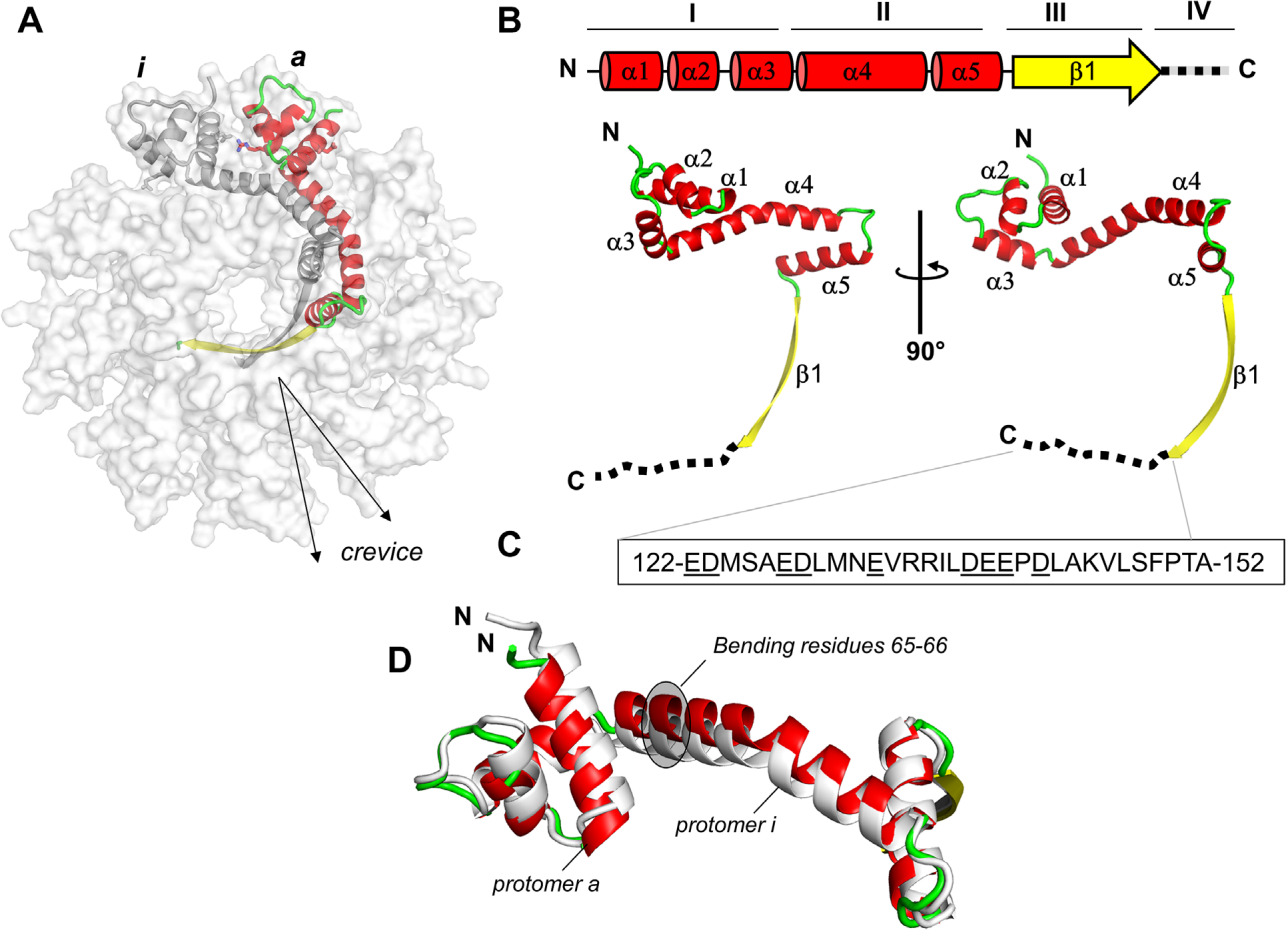
Organization of PaP3 TerS protomer. (**A**) Ribbon diagram of PaP3 TerS protomer colored by secondary structure elements with α-helices, β-strands and random coil in red, yellow and green, respectively, overlaid to a semi-transparent solvent surface of the nonamer. (**B**) Topological diagram and ribbon representation of the TerS protomer, which contains five α-helices and one β-strand. Residues 122–152, invisible in the electron density, are dashed in black. (**C**) Amino acid sequence of TerS C-terminal tail spanning residues 122–152; underlined are all acidic residues. (**D**) Secondary structure superimposition of protomer *a* (red, green, and yellow) and protomer *i* (gray), which are the most dissimilar in the oligomer (RMSD ∼1.67 Å).

The tertiary structure of PaP3 TerS protomer resembles an ‘L’ (Figure 3A), and in the oligomer, all protomers stand parallel to the 9-fold axis running along the central channel. The protomer can be divided into four regions (Figure 3B): (i) an N-terminal α-helical core formed by 3 α-helices (α_1_–α_3_) that protrude outwards, decorating the entire perimeter of the ring (res. 18–63) and that includes the HTH putatively involved in DNA-binding; (ii) an α-helical hairpin (α_4_–α_5_) built by two long helices running orthogonal to each other (res. 64–105); (iii) a β-strand (res. 107–121) that forms the 9-stranded β-barrel; (iv) an acidic C-terminal tail (res. 122–152) not visible in the crystal structure (Figure 3C), though present in the crystallized construct. This tail is likely disordered in the crystal structure or fails to obey 9-fold symmetry (Figure 1A). Helices α4–α5 and strand β1 form the oligomerization core, while the helix α4 also connects to the N-terminal HTH that projects outward. In agreement with the high B-factor of this area of the structure, DynDom (71) predicts a flexible hinge between residues ^65^FI^66^ of helix α4 (highlighted in gray in Figure 3D) that allow for 16.5° rotation of the HTH with respect to α4. The paucity of bonding interactions between neighboring HTH domains and the flexible articulation to helix α4 suggest HTHs can move laterally, as well as above and below the plane formed by the apical gear-like ring. Not surprisingly, the nine protomers show significant variation in the atomic position of the HTHs with chains *a*-*i* being the most dissimilar (RMDS ∼1.67 Å) (Figure 3D). As a result, the crevice between protomers (Figure 3A) varies throughout the oligomer and is deeper for protomers where R56 and D21 do not form a salt bridge. The same intramolecular plasticity observed in PaP3 likely exists in the TerSs from NV1 and LUZ24. The latter, in particular, had poorly defined spokes even at low-resolution in a symmetrized map (Supplementary Figure S6). As seen for the *Bacillus* TerS (34) (Supplementary Figure S4A), loose bonding interactions between HTHs and the oligomerization core result in a poorly symmetric oligomer that breaks strict 9-fold rotational symmetry.

### Solution structure of PaP3 and NV1 TerSs

**Figure 4.**
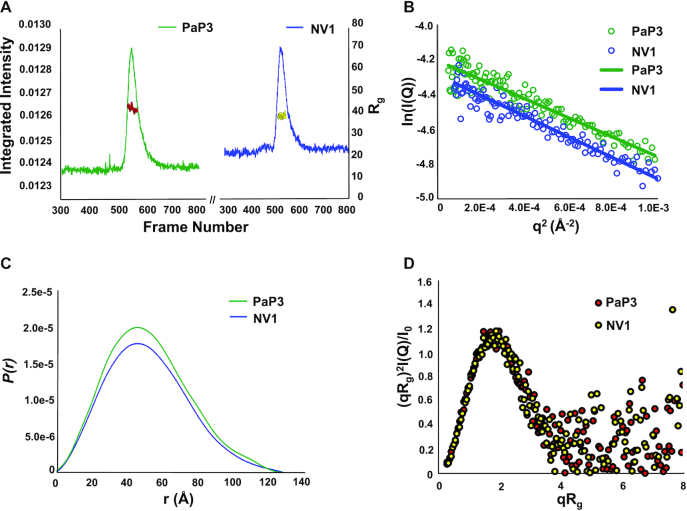
SEC-SAXS analysis of PaP3 and NV1 TerSs. (**A**) SEC-SAXS profile of PaP3 TerS at 1.7 mg ml^−1^ and NV1 TerS at 2.0 mg ml^−1^ measured in 20 mM Tris–HCl pH 8.0, 0.15 M NaCl, 2.5% glycerol and 0.5 mM TCEP at 4°C. The red dots and yellow dots indicate R_g_ values (on the *y*-axis) corresponding to frames (*x*-axis) for PaP3 TerS and NV1 TerS, respectively. (**B**) PaP3 TerS Guinier plots calculated from averaging buffer-subtracted scattering intensities from frames (495–514) and the sample peak (547–564) (green dots). The PaP3 TerS coefficient of determination for line of best fit, *R*^2^, is 0.8942 (green line). NV1 TerS Guinier plots calculated from averaging buffer-subtracted scattering intensities from frames (280–299) and the sample peak (523–542) (blue dots). The NV1 TerS coefficient of determination for line of best fit, *R*^2^, is 0.9267 (blue line). (**C**) P*(r)* function (**D**) and dimensionless Kratky plot calculated from SEC-SAXS data.

To shed light on the solution structure of TerS, we carried out size exclusion chromatography coupled with small-angle X-ray scattering (SEC-SAXS) (72) in a concentration range between 1.7–2.0 mg ml^−1^, about one-fifth of the concentration used for crystallization (Table 3 and Figure 4A). Both PaP3 and NV1 TerSs gave good SEC-SAXS profiles suitable for biophysical analysis, whereas LUZ24 TerS displayed a strong tendency to aggregate and was not pursued further. SEC-SAXS of PaP3 and NV1 TerS revealed a radius of gyration (R_g_) of 41.44 ± 0.40 Å and 41.33 ± 0.43 Å, respectively, (Figure 4B and Table 3), which is larger than the calculated crystal structure R_g_ values of 33.84 Å and 33.32 Å, respectively. The PaP3 and NV1 Guinier plots revealed a featureful scattering curve characterized by ‘humps’ at mid*-q* values, as expected for a hollow molecule (Supplementary Figure S8A). The distance distribution function *P(r)* calculated from SAXS data indicates a maximum diameter *D*_max_ ∼131 Å for both PaP3 and NV1 TerS, also about 25% larger than the maximum width of the crystal structure (∼105 Å) (Figure 4C). The Volume of Correlation (Vc) mass calculated from SAXS data for both PaP3 and NV1 TerS is 145.9 ± 14.6 kDa and 147.1 ± 14.7 kDa, respectively, which are close to the expected M.W. of a nonameric ring (M.W. ∼153.1 kDa for PaP3 and ∼159.6 kDa for NV1). The dimensionless Kratky plots have a peak at qRg = √3 and a peak height of 3/*e*, indicating a globular shape, but also falls to zero consistent with a folded structure (Figure 4D) (73).

**Table 3.** SEC-SAXS data collection and refinement statistics

SEC-SAXS	PaP3 TerS	NV1 TerS
Instrument	ID7A1	ID7A1
Wavelength (Å)	1.234	1.234
Exposure time (s)	0.5	0.5
Protein Concentration (mg ml^−1^)	1.7	2.0
Temperature (K)	277	277
Radius of Gyration, R_g_* (Å)	41.44 + 0.40	41.34 + 0.43
Maximum Diameter, *D*_max_	131	131
Volume of correlation M.W./Theoretical M.W. (kDa)	145.9/153.0	147.1/159.7
	**Software employed**	
Primary data reduction	RAW version 1.6.3	
Data processing	ATSAS	
*Ab initio* analysis	DENSS	
Validation and averaging	EMAN2	
Rigid-body refinement	*Phenix.real_space_refine*	
Computation of model intensities	FoxS	
3D-graphics representations	Chimera	

*Rg was determined from Guinier plot.

**Figure 5.**
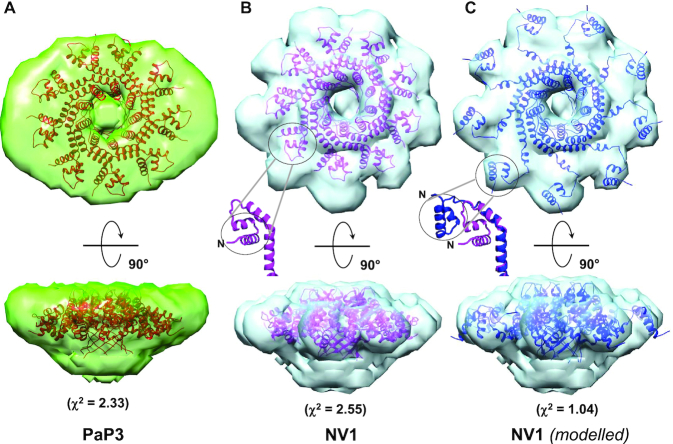
Solution structure of PaP3 and NV1 TerSs. *Ab initio* SEC-SAXS electron density of PaP3 (**A**) and NV1 (**B**) TerS at FSC resolution of 33.3 Å and 38.1 Å, respectively (Supplementary Table S1). Ribbon diagrams of the crystallographic models are overlaid to the electron densities with Pap3 TerS in orange and NV1 TerS in purple. The black circle in panel B and its relative zoomed-in insertion indicates one HTH in the close conformation. (**C**) The modeled structure of NV1 TerS with open HTHs is overlaid to the SAXS density. The zoomed-in insertion shows a superimposition of HTHs in the closed (purple) versus open (blue) conformations. The agreement between solution and crystal structures was calculated using the FoxS server (see Supplementary Figure S8).

To visualize the structural organization of the PaP3 and NV1 TerS in solution, we calculated an electron density from solution scattering data using DENSS (64) (Table 3). The SAXS density had an estimated Fourier Shell Correlation (FSC) resolution of 57.9 Å and 56.7 Å for PaP3 and NV1 TerS, respectively, which sharply improved after applying 9-fold symmetry to 33.3 Å and 38.1 Å, respectively (Supplementary Table S1). In contrast, using 10- or 8-fold rotational symmetry did not significantly improve the FSC resolution, supporting the notion PaP3 and NV1 TerSs exist in solution as nonamers (Figure 5A and B). The SAXS electron density is shaped like a ‘donut’ with a tapered end that fits the β-barrel, a disk-like upper domain ∼140 Å in diameter that includes a central channel of ∼20 Å. The crystallographic structure of PaP3 TerS was docked inside the 9-fold averaged density and rigid-body refined against the SAXS density, revealing good, but not perfect agreement between solution and crystalline states (*χ*^2^ = 2.33) (Figure 5A and Supplementary Figure S8B). Similarly, the NV1 TerS crystal structure docked into the 9-fold electron SAXS density revealed modest agreement between solution and crystal structure (*χ*^2^ = 2.55), despite the exceptional quality of this SAXS density (Figure 5B and Supplementary Figure S8C). In both cases, the solution structure of TerS had a wider outer diameter than the crystal structure and showed extra density for the missing C-terminal tail. This discrepancy was also revealed by the difference in *D*_max_ between *P(r)* function and the width of crystal structures (Figure 4C). The SAXS density has pronounced density for nine outer lobes, which is strikingly different from the closed conformation of HTHs seen in the crystal structure. We then modeled an open conformation of the HTHs that best fits the SAXS density by subjecting HTH helix α1 and α2 to a ∼60° rigid-body rotation around the helix α3. The structure of TerS with outward swung HTHs improved to the correlation with SEC-SAXS data to an impressive *χ*^2^ = 1.04 (Figure 5C and Supplementary Figure S8D). Thus, X-ray scattering and modeling data suggest the open conformation of TerS is more populated in solution than the closed state captured in the crystal structure.

### Mechanisms of DNA-binding

**Figure 6.**
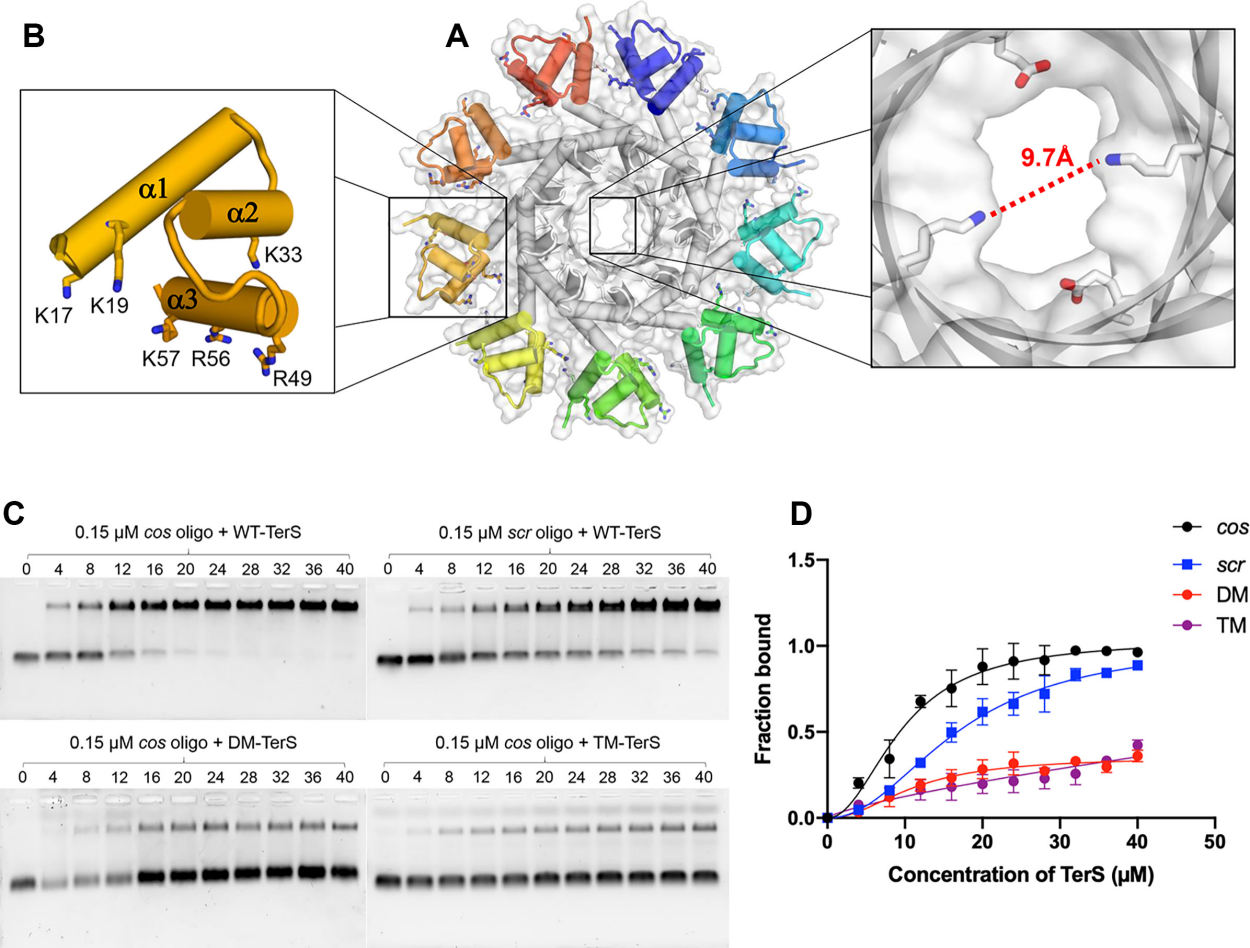
DNA-binding activity of PaP3 TerS. (**A**) Solvent surface view of PaP3 TerS with a magnified view of the central channel, which has a minimum diameter of 9.7 Å, too narrow to accommodate dsDNA. (**B**) Zoom-in view of the N-terminal HTH domain; basic residues, putatively involved in DNA-binding, are shown as sticks. (**C**) Native agarose gel electrophoresis of PaP3 WT-TerS binding to Cy3-*cos (top left) and Cy3-scr* DNA (top right) as well as DM-TerS (bottom left) and TM-TerS (bottom right) binding to Cy3-*cos*. In all gels, a fixed concentration of *cos* DNA was titrated against 0–40 μM of TerS. (**D**) Quantification of band shift data in panel C. The error bar represents the standard deviation calculated from three independent gels.

PaP3 TerS was previously shown to have DNA-binding activity *in vitro* (48), but the specific residues implicated in DNA-binding are unknown. The crystal structures of PaP3 and NV1 TerSs presented in this paper revealed a solvent-filled channel of 9.7–15 Å diameter (Figure 6A). This channel is too small to accommodate dsDNA, ruling out the viral genome threads through the central channel of TerS during packaging (74). To decipher the residues involved in DNA-binding, we generated alanine point mutations in each of the basic residues exposed in HTH helices α1, α2 and α3 (Figure 6B). The first mutant (named ‘Double Mutant’ or DM) had K17 and K19 in helix α1 mutated to alanine. The second mutant (named ‘Triple Mutant’ or TM) had K33 in helix α2 in addition to the two mutations in DM. We also generated a polyAla mutant (pAla-TerS) that had six basic residues in the HTH, namely, K17/K19 in helix α1, K33 in helix α2 and R49/R56/K57 in helix α3 (Figure 6B). Both DM- and TM-TerS showed as monodisperse by SEC and migrated indistinguishably from the WT-TerS (Supplementary Figure S9A). In contrast, the pAla-TerS was slightly shifted to the right, possibly consistent with a smaller oligomer or an improperly assembled species (Supplementary Figure S9A). We therefore decided to use only DM- and TM-TerS for DNA-binding studies and omitted pALA-TerS. We also generated ΔC122-TerS, which lacks the C-terminal tail proven essential for DNA- (30,31) and TerL-binding (31,75) in P22, but not crucial for DNA-association in SPP1 TerS (23,76). The C-terminal tail is remarkably acidic in PaP3, with seven Asp and Glu residues between amino acids 122–137 (underlined in Figure 3C). ΔC-TerS migrated slightly smaller than WT-TerS by SEC, consistent with the deletion of ∼270 amino acids (Supplementary Figure S9B).

We used an EMSA on agarose gel to investigate the DNA-binding activity of PaP3 TerS. WT-TerS from peak 2 (Figure 1A) was incubated with a Cy3-fluorescently labeled 20-mer oligonucleotide containing the PaP3 *cos* sequence (48) or a scrambled DNA oligonucleotide. After electrophoretic separation, both free DNA and TerS:DNA complexes were visualized using the fluorescence signal of Cy3 and quantified (Figure 6C and D), which revealed PaP3 TerS binds the *cos* sequence with significantly higher affinity than scrambled DNA. Mutations in the HTH helix α1 (DM-TerS) reduced binding to *cos* (Figure 6C- and D) by over 70%, confirming this region of the protein is responsible for DNA-binding activity *in vitro*. The association between TM-TerS and *cos* was also majorly disrupted, even more than DM, suggesting the majority of binding determinants for DNA are in the first two helices α1 and α2. In contrast, ΔC122-TerS had similar DNA-binding properties for *cos* DNA as WT-TerS, both on agarose and acylamide gels (Supplementary Figure S10). Thus, PaP3 C-terminal tail does not appear to play a role in DNA-binding. This tail is also not involved in terminase association as PaP3 TerS and TerL do not stably associate together, at least when expressed in bacteria (Supplementary Figure S11).

## DISCUSSION

*Pseudomonas aeruginosa* is the most common gram-negative bacterium found in hospital-acquired infections. It is responsible for 30% of deaths caused by pneumonia and septicemia and is a significant cause of concern for respiratory tract infections. There is an increasing interest in understanding the biology of *Pseudomonas*-phages, which is fueled by their utilization in phage therapy (77,78). However, *Pseudomonas-*phages remain significantly less studied than classical coliphages, and the mechanisms of genome-packaging are mostly unexplored. In this paper, we examined three evolutionarily related TerSs from *Pseudomonas*-phages isolated from hospital sewage. Using hybrid biophysical methods, we shed light on the structure and conformational dynamics of the small terminase subunit, as it relates to its DNA-binding activity.

### Conserved nonameric quaternary structure

The structures of PaP3, NV1 and LUZ24 TerSs presented in this paper expand the repertoire of viral terminase subunits characterized at the molecular level in the last decade. By combining fractionation on heparin with rigorous biophysical analysis, we determined TerS is polymorphic in solution but always folds into a nonamer in the high affinity heparin-binding conformation, which we hypothesize represents the high-affinity DNA-binding state of this protein. The crystal structure of PaP3 presented here represents the first complete 3D-structure for a TerS from a *cos*-packager. The structures of PaP3, NV1 and LUZ24 TerSs together with six previously characterized homo-oligomeric TerSs from phages P22 (10,30–31,70,79), SF6 (34), G20c (PDB 4XVN), P76–26 (25), HK97 (80) and a prophage of *Bacillus Cereus* (PDB 2AO9) bring the total number of nonameric TerSs to nine. Thus, we propose TerS is always nonameric in the high-affinity DNA-binding conformation (e.g. this hypothesis does not include phage λ small terminase subunit gpNu1 that exists in a stable hetero-oligomer with the TerL gpA (81)). Because of the polymorphic self-assembly described in this paper, TerS can potentially crystallize in different oligomeric states. By analogy, the portal protein forms polymorphic rings *in vitro* (82–84) but still exists as a dodecamer in virion (84). There are only two exceptions: Sf6 TerS, which crystallized as an octamer (32), and TerS from T4-like phages that form a mixture of decamers and undecamers, both in crystal and in solution (85,86). Sf6 is a headful packager similar to P22 (75), and its TerS recognizes a specific *pac* site. It seems unlikely Sf6 reinvented the small terminase subunit in a different oligomeric state. Future studies will have to determine if the octameric structure of Sf6 TerS visualized in the crystal (32) also exists in solution and corresponds to a high-affinity DNA-binding species. More complex is the case of T4-like phages (74), which package genomes by a variant of the headful packaging mechanism whereby no *pac* site is recognized, and packaging initiates randomly. In these phages, TerS folds into a simple helical hairpin that lacks a canonical HTH (85,86). It is possible the oligomeric conformation of T4 TerS simply functions by creating an outer surface for DNA adsorption (74), which is successful because T4-phages degrade the host DNA, ensuring that only their DNA is available for packaging. Thus, though a nonameric quaternary structure is emerging as the most common and perhaps universal fold for TerSs that bind DNA via an HTH motif, different mechanisms of DNA recognition, and possibly quaternary structures, may also exist in nature.

### A model for sequence-specific DNA recognition

**Figure 7.**
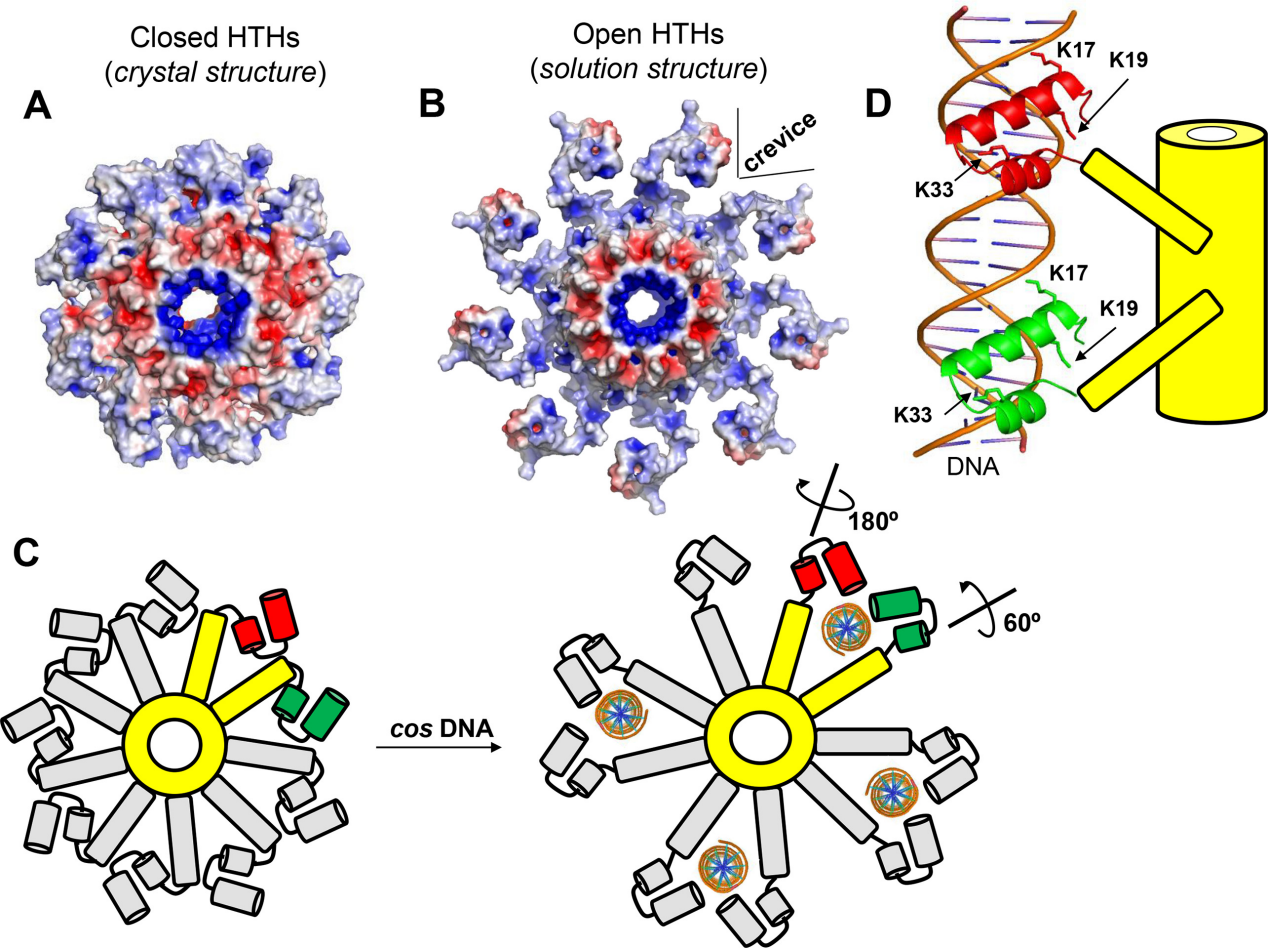
Proposed model for TerS recognition of packaging initiation sites by DNA-interdigitation. (**A**) Electrostatic surface potential of TerS with closed HTHs observed in the crystal structure and (**B**) the open conformation of TerS modeled based on the solution structure. In both panels, the electrostatic surface potentials range between −5 kT/e representing negative charges (colored in red) and +5 kT/e representing positive charges (colored in blue). (**C**) Schematic top-view of PaP3 TerS: the oligomerization core and two nearby protomers are colored in yellow while the reminder seven protomers are in gray. The HTH helices α1–α2 are colored in green (protomer #1) and red (protomer #2). Four ds-DNA molecules are modeled as interdigitated by four pairs of TerS protomers. (**D**) Schematic representation of a 20-mer DNA oligonucleotide laterally interdigitated by two subunits of TerS whose HTH helixes α1 and α2 are color-coded as in panel (C).

Despite decades of research, the mechanisms of TerS binding to packaging initiation sites are not fully understood. Two mainstream models have been proposed: the ‘nucleosome-model’ (30,32,34,39,86), whereby DNA wraps around the outer rim of TerS, and the ‘threading-model’ that contemplates DNA going through the TerS central channel (31). Whereas the threading model is disproved for T4 (74) and is structurally unfeasible for TerSs that possess a narrow central channel like PaP3 TerS, the nucleosome-model lacks strong experimental evidence. TerSs from *pac* packagers bind DNA weakly and non-specifically *in vitro* (25,30,32,35), unlike histones that bind tightly to DNA via electrostatic interactions. Nevertheless, despite mechanistic differences, both models fall short in explaining the *in vivo* specificity of TerS for packaging initiation sites, which is the most important biological property of this protein. A specificity without which genome packaging would not proceed in most viruses (27). DNA wrapping around TerS is a binding mode used by sequence-independent proteins like histones that lack nucleobase-specificity. Even less specific is threading the double helix through the central channel of TerS, which is intrinsically sequence-independent. The biophysical analysis of three TerSs presented in this paper paves the way to decipher TerS specificity for packaging initiation sites. First, we demonstrate that the central channel of PaP3 TerS has a diameter of ∼9.7 Å (Figure 6A), too small to accommodate hydrated dsDNA, as previously observed for Sf6 (32) and SF6 (34) TerSs, which rules out a threading model for DNA-passage (74). Second, PaP3 TerS was never found as a dimer of nonamers, ruling out a twin ring model proposed for T4 (87). Third, unlike P22 (30,31), removing the C-terminal tail did not disrupt DNA-binding (Supplementary Figure S10), as observed for SPP1 TerS (23,76). Fourth, we failed to detect a physical association between PaP3 terminase subunits *in vitro* (Supplementary Figure S11), suggesting TerL and TerS may not form a stable complex during genome-packaging, unlike λ (42) and P22 (10). Fifth, disrupting basic residues in the HTH-domain abolished sequence-dependent binding to a dsDNA oligonucleotide, confirming the HTH is the primary determinant for DNA-binding, as shown for phage λ (39), also a *cos* packager, Sf6 (33,75) and SF6 (35), which are *pac* packagers. Sixth, we identified intrinsic structural plasticity in the way HTHs connect to the oligomerization core of PaP3 TerS. Even more extreme flexibility was observed in SF6 TerS (34), where the connections between HTHs and the oligomerization core were invisible in the crystal structure. HTHs interact loosely at TerS perimeter due to the scarcity of bonding interactions between neighboring HTHs and the intrinsic ring asymmetry, which, we propose, is a direct consequence of adopting a nonameric quaternary structure. While an even number of subunits (e.g. an octamer or a decamer) results in identical contacts between adjacent protomers, which locks a molecule into one conformation, a nonamer lacks perfect complementarity of binding interfaces, which can result in a non-equivalent protomer having a higher degree of freedom. This is perhaps the reason why TerSs tend to be nonameric, to promote lateral movement of HTHs. In agreement with this idea, SEC-SAXS revealed significant differences between crystal and solution structures of PaP3 and NV1 TerSs. The closed yet asymmetric arrangement of HTHs seen in the crystal structure (Figure 7A) is not representative of the solution structure of this molecule that we extrapolated by modeling an open conformation of the HTHs in the SAXS density (Figure 7B).

How does conformational plasticity in HTHs affect DNA-recognition in PaP3? We propose the open conformation of TerS generates a unique binding crevice for DNA at the interface between two protomers (Figure 7B) that allows side chains from two HTHs to deepen inside the major groove of DNA. In this regard, it should be noted that the three helices of an HTH serve different roles in DNA recognition (88). The first helix (α1) binds DNA nonspecifically, facilitating the correct positioning of the second helix (α2), which binds to a consensus sequence along the major groove of DNA (89). The turn motif typically contains additional amino acids that can contact DNA individually and potentially extend into longer ‘winged’ turns. The third helix (α3), which is not always present, plays a minor role in DNA-binding (90). In the crystal structure of PaP3 TerS, helix α1 exposes K17 and K19 that decorate the outer rim of the terminase, generating the first surface of interaction with DNA. This is supported by our EMSA data, whereby mutations of K17 and K19 abrogated over 70% binding to DNA (Figure 6C and D). This loss of function was further reinforced by mutation of K33 in helix α2 that disrupted DNA-binding by nearly 80%. The closed conformation of TerS seen in the crystal structure (Figure 7A and C) is generated by a rigid-body rotation of the α1–α2 hairpin around helix α3 (Figure 5B and C). Indirect evidence for the flexibility of this hairpin and ability to extend outward were provided by the subunit plasticity seen in the crystal structure (Figure 2B) and inferred by SAXS modeling (Figure 5B and C). We propose K17/K19 and K33 specifically recognize a *cos* site by making sequence-specific contacts with DNA. This recognition requires laterally extended HTHs with the helical hairpin α1–α2 expanding outward, as modeled in Figure 5C. In this binding mode, which we will refer to as ‘lateral DNA-interdigitation,’ dsDNA threads between two nearby HTHs that insert basic side chains into the major groove, at the outer ends of an ∼20-bps *cos* site (Figure 7C and D). TerS would bind *cos* DNA specifically by using two juxtaposed HTH motifs emanating from adjacent subunits. This model implies that while one HTH simply rotates by 60° (colored in green in Figure 7C and D), the HTH from a neighboring subunit would make a much larger rotation (∼180°) around the flexible region between helices α2 and α3 (colored in red in Figure 7C and D), allowing two neighboring HTHs to bind DNA in a dimeric fashion. This DNA recognition mode resembles how HTHs from the homodimeric Cro protein from bacteriophage ʎ (91) bind DNA (Supplementary Figure S12). In the Cro protein:DNA complex, each subunit contributes one HTH motif, which binds DNA in the major groove with both helices α2 and α3. Alternatively, it is possible that one TerS HTH does not make a full rotation to recognize DNA inside a binding crevice, but two HTHs from nearby subunits bind opposite DNA sites, using residues K17/K19 from helix α1 of one subunit and K33 from helix α2 of another HTH. This recognition could seemingly facilitate recognition of repetitive *cos* sub-site within a more extended sequence, as suggested for the λ gpNu1 dimer bound to DNA (39) and be facilitated by DNA bending (39), or proteins that induce DNA tertiary structures (92).

DNA-interdigitation could, in principle, allow more copies of TerS to simultaneously bind DNA, as observed in high order complexes of TerS with DNA reported for SPP1 (28), λ (39) and Sf6 (32). Similarly, TerS could bind to a discrete number of dsDNA sequences (e.g. four, as in Figure 7C), possibly stabilizing a DNA loop or synapse (87). Besides, this binding mode can potentially explain the involvement of the C-terminal tail reported for P22 (31). A flexible tail projecting from the bottom of the β-barrel could extend laterally and insert into the groove of DNA held by two HTHs. Finally, lateral DNA-interdigitation explains how a substoichiometric number of C-terminal tails in P22 TerS can recruit TerL while DNA is bound to TerS (10), forming a complex that binds to the portal vertex. The model presented in this paper for PaP3 TerS does not clearly apply to the thermophilic phage P76–26, whose TerS was recently determined using cryo-electron microscopy (25). In this TerS from a phage adapted to extreme conditions, the nonameric ring has HTHs rigidly held in an orientation distinct from what is seen in PaP3 TerS and most other known TerSs. The rigidity of HTHs in a thermophilic phage reveals key differences with mesophilic counterparts like PaP3 that reinforces the notion TerSs have diversified significantly regardless of a somewhat similar fold to fit different packaging strategies and environmental conditions.

In conclusion, this paper provides novel insights into the structure and conformational plasticity of *Pseudomonas*-phage TerS. These ideas led us to propose a structural model for sequence-specific binding to packaging initiation sites of general applicability to TerSs from other viruses.

## DATA AVAILABILITY

DynDom is a web server for protein domain movement analysis

http://dyndom.cmp.uea.ac.uk/dyndom/runDyndom.jsp

PDBePISA is an interactive tool for the exploration of macromolecular interfaces.

https://www.ebi.ac.uk/pdbe/prot_int/pistart.html

FoXS is a web server for fast SAXS profile computation with Debye formula.

https://modbase.compbio.ucsf.edu/foxs/

PDB2PQR is a web server that enables a user to convert PDB files into PQR files.

http://nbcr-222.ucsd.edu/pdb2pqr_2.1.1/

DALI is a web server for comparing protein structures in 3D.

http://ekhidna2.biocenter.helsinki.fi/dali/

Atomic coordinates and structure factors have been deposited with the Protein Data Bank under accession codes 6W7T and 7JOQ.

## Supplementary Material

gkaa866_Supplemental_File
