# Supplementary material for: Biophysical analysis of Pseudomonas-phage PaP3 small terminase suggests a mechanism for sequence-specific DNA-binding by lateral interdigitation
Source: Nucleic Acids Res. 2020 Oct 30;48(20):11721–36. doi: 10.1093/nar/gkaa866 (PMC7672466; doi:10.1093/nar/gkaa866)
Supplement: gkaa866_Supplemental_File [file gkaa866_supplemental_file.pdf]

SUPPLEMENTARY DATA

Supplementary Table S1
Supplementary Figures S1-S12

Biophysical analysis of *Pseudomonas*-phage PaP3 small terminase suggests a mechanism for sequence-specific DNA binding by lateral interdigitation

Marzia Niazi[1,§], Tyler J. Florio[1,§], Ruoyu Yang[1,§], Ravi K Lokareddy[1,§], Nicholas A. Swanson[1,§], Richard E. Gillilan[2], and Gino Cingolani[1,*]

[1] Department of Biochemistry and Molecular Biology, Thomas Jefferson University, 1020 Locust Street, Philadelphia, PA 19107, USA
[2] Macromolecular Diffraction Facility, Cornell High Energy Synchrotron Source (MacCHESS), Cornell University, 161 Synchrotron Drive, Ithaca, NY 14853, USA

* To whom correspondence should be addressed. Tel: 01 (215) 503 4573; Fax: 01 (215) 464 4595; E-mail: gino.cingolani@jefferson.edu

[§] Joint First Authors

	**Applied Symmetry Operator (fold)**	**Fourier Shell Correlation Resolution (Å)**
**PaP3 TerS**	1	57.9
	8	40.3
	**9**	**33.3**
	10	43.4
**NV1 TerS**	1	56.7
	8	47.6
	**9**	**38.1**
	10	45.5

Supplementary Table S1. How the Fourier Shell Correlation (FSC) resolution of PaP3 and NV1 SAXS maps varies by applying alternative symmetry operators.

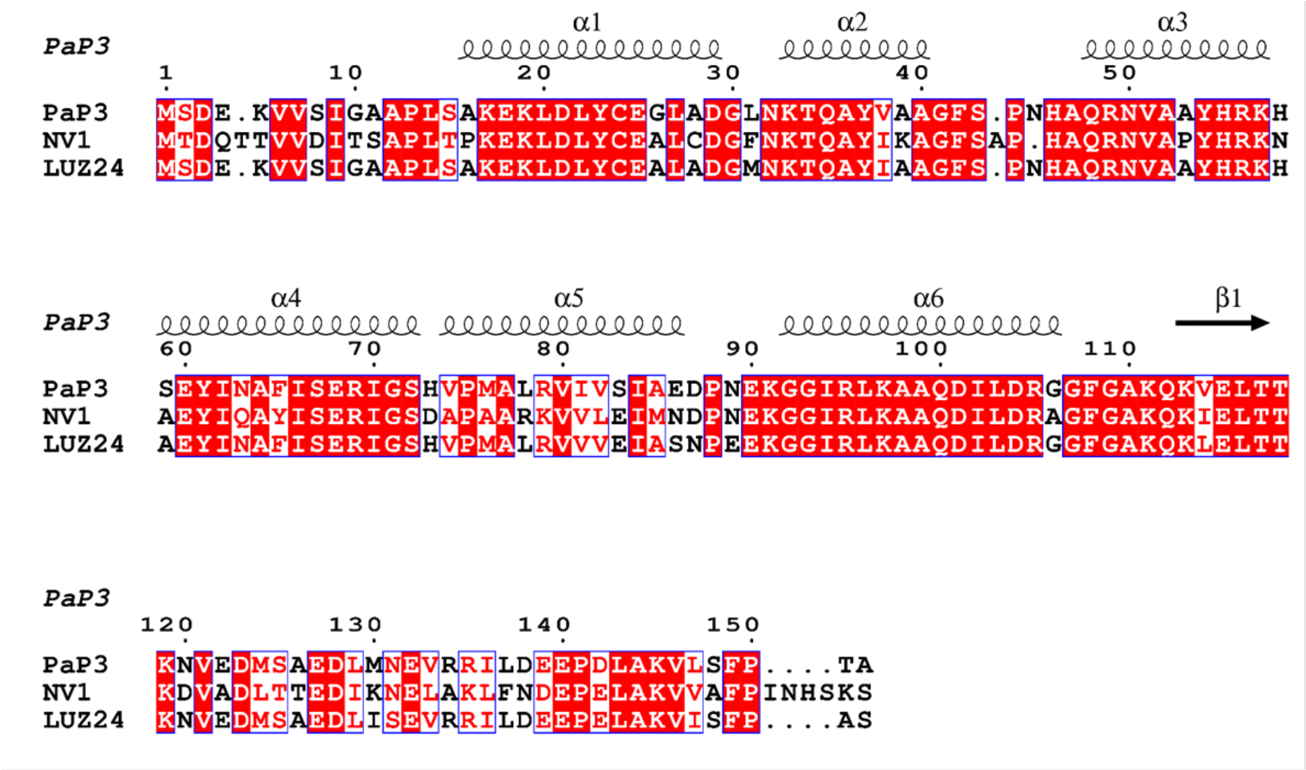

Supplementary Figure 1. Sequence alignment of TerS from *Pseudomonas* phages PaP3, NV1, and LUZ24. The sequence alignment was done with ClustalW (1) and displayed using Esprit3 (2).

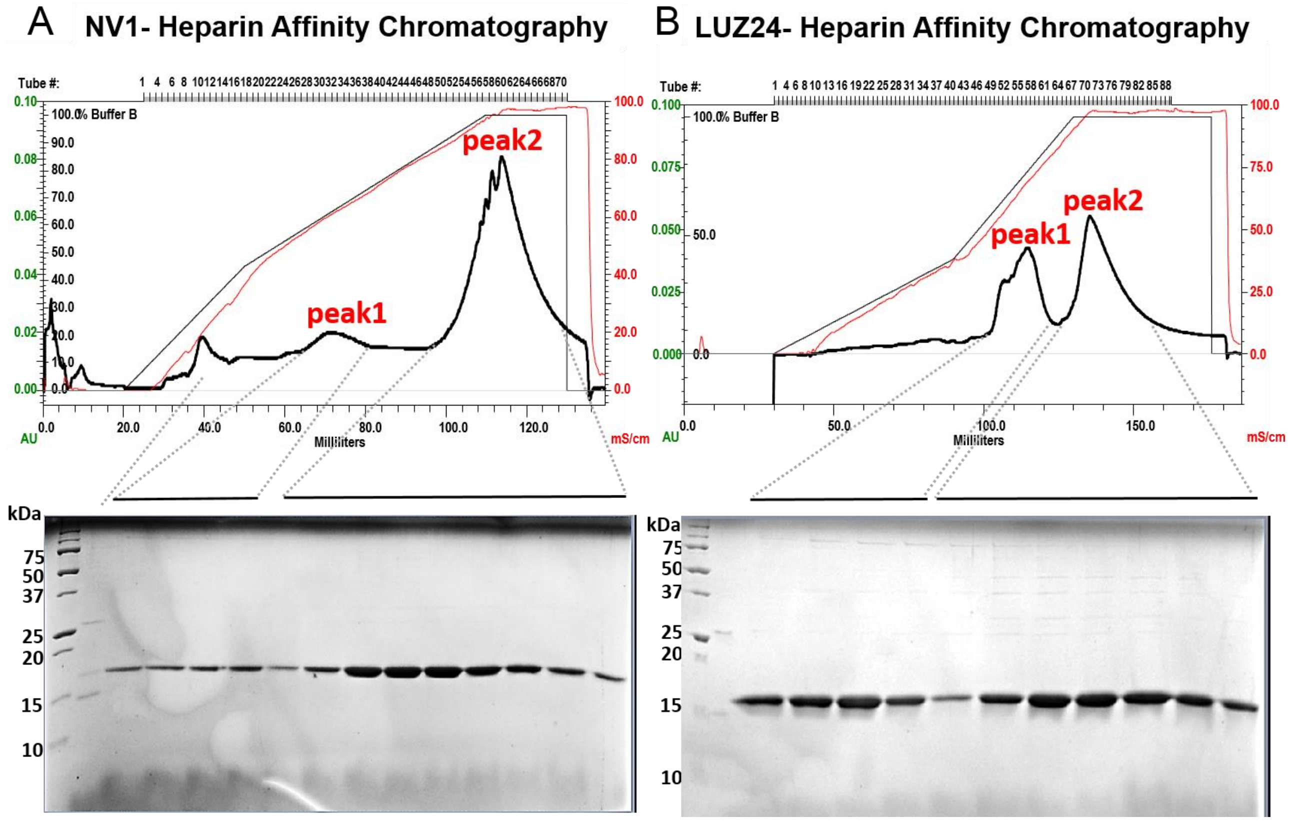

Supplementary Figure 2. Heparin purifications of LUZ24 and NV1 TerSs. (**A**) Heparin-affinity chromatography yields two species: peak 1 and peak 2 in the case of LUZ24 and predominantly peak 2 in the case of NV1 TerS. (**B**) SDS-PAGE of corresponding fractions from heparin purification.

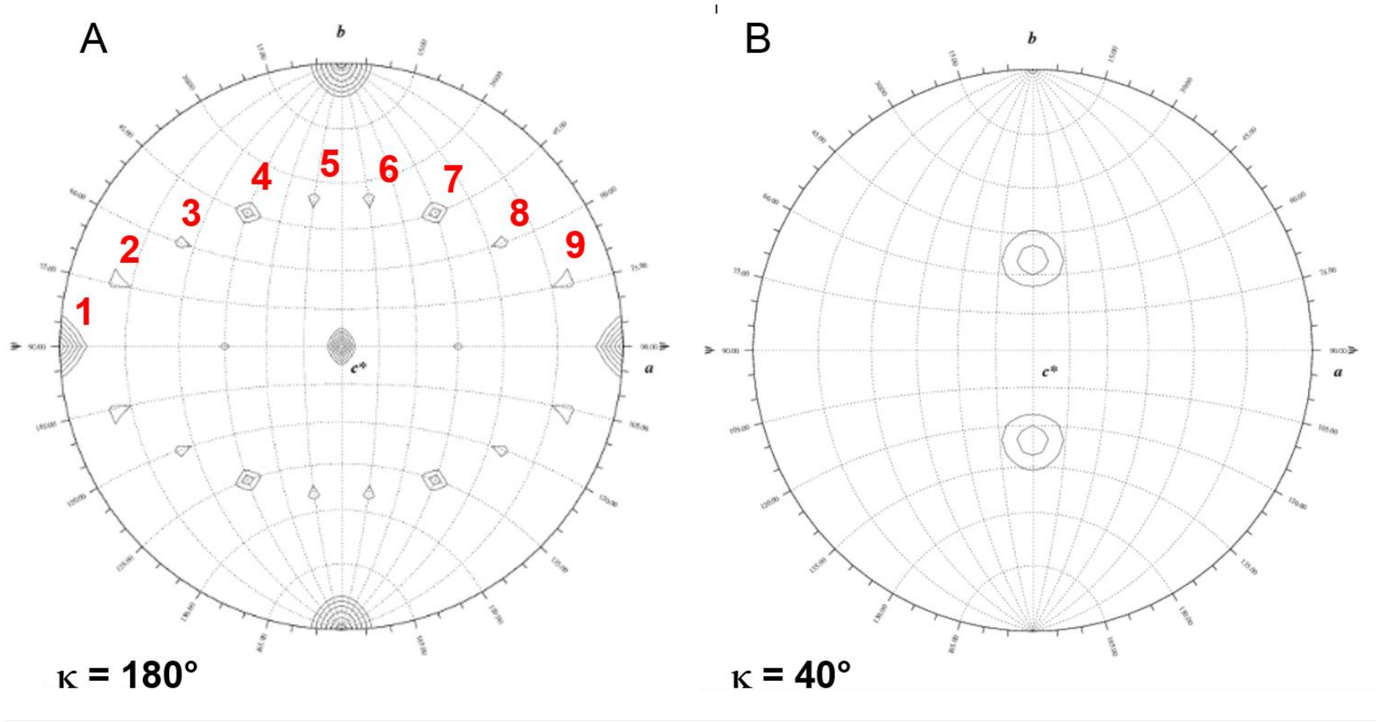

Supplementary Figure 3. PaP3 TerS crystallizes as a nonameric assembly in the asymmetric unit. Self-rotation function computed using diffraction data between 12-3.5 Å with the k vector fixed at 180° (**A**) and 40° (**B**). The plots suggest the existence of nine 2-fold rotation axes (in red) and one 9-fold rotation axis, which is consistent with a nonamer in the asymmetric unit. The self-rotation function was computed using GLRF (3).

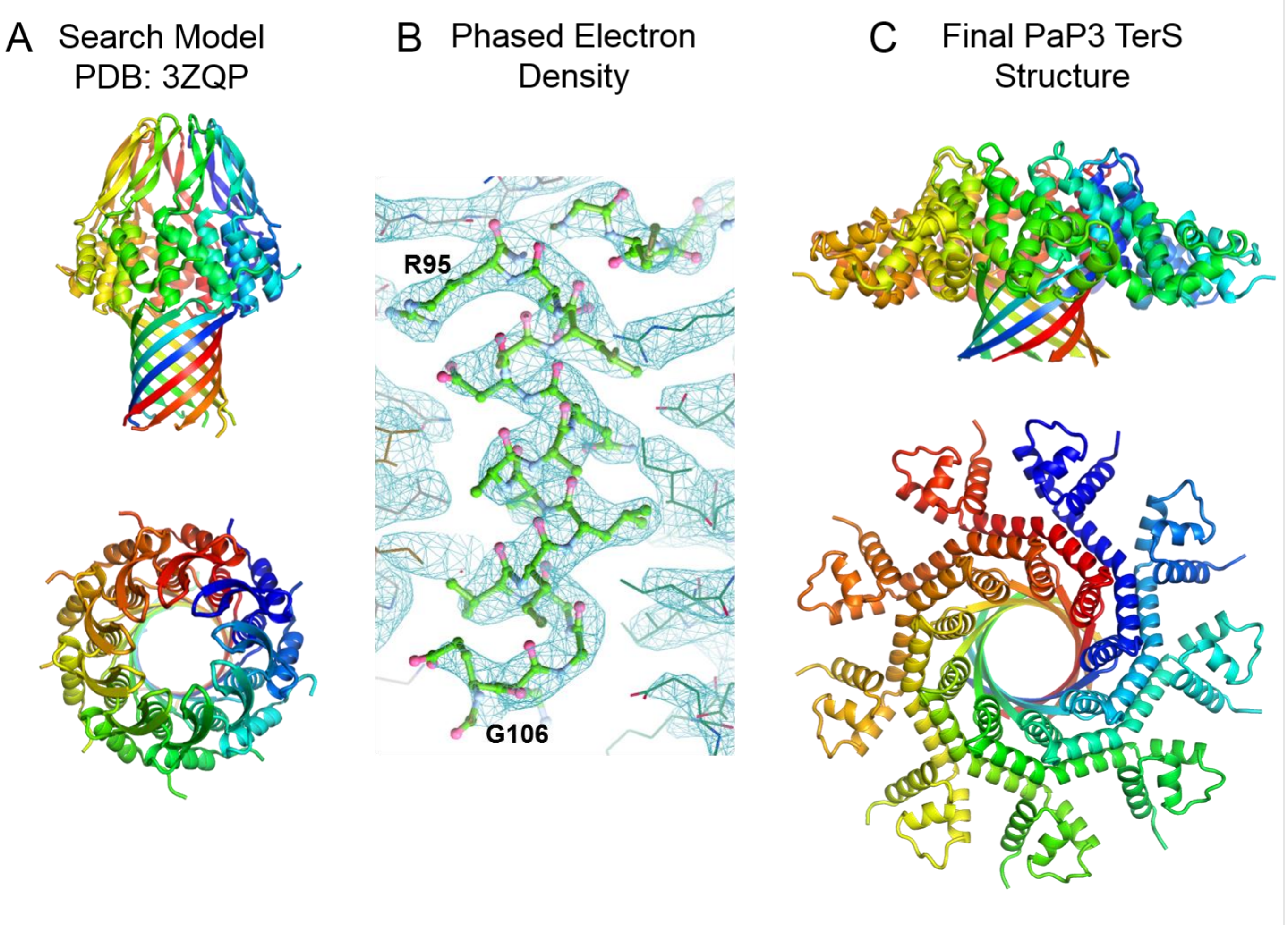

Supplementary Figure 4. Phasing PaP3 TerS. (**A**) Oligomerization core of SF6 (PDB: 3ZQP) used as a search model for Molecular Replacement (MR). (**B**) Experimental 2Fo-Fc electron density map overlaid to the final atomic model (residues 95-106). (**C**) Ribbon diagram of the PaP3 TerS structure shown in scale with the search model in panel A.

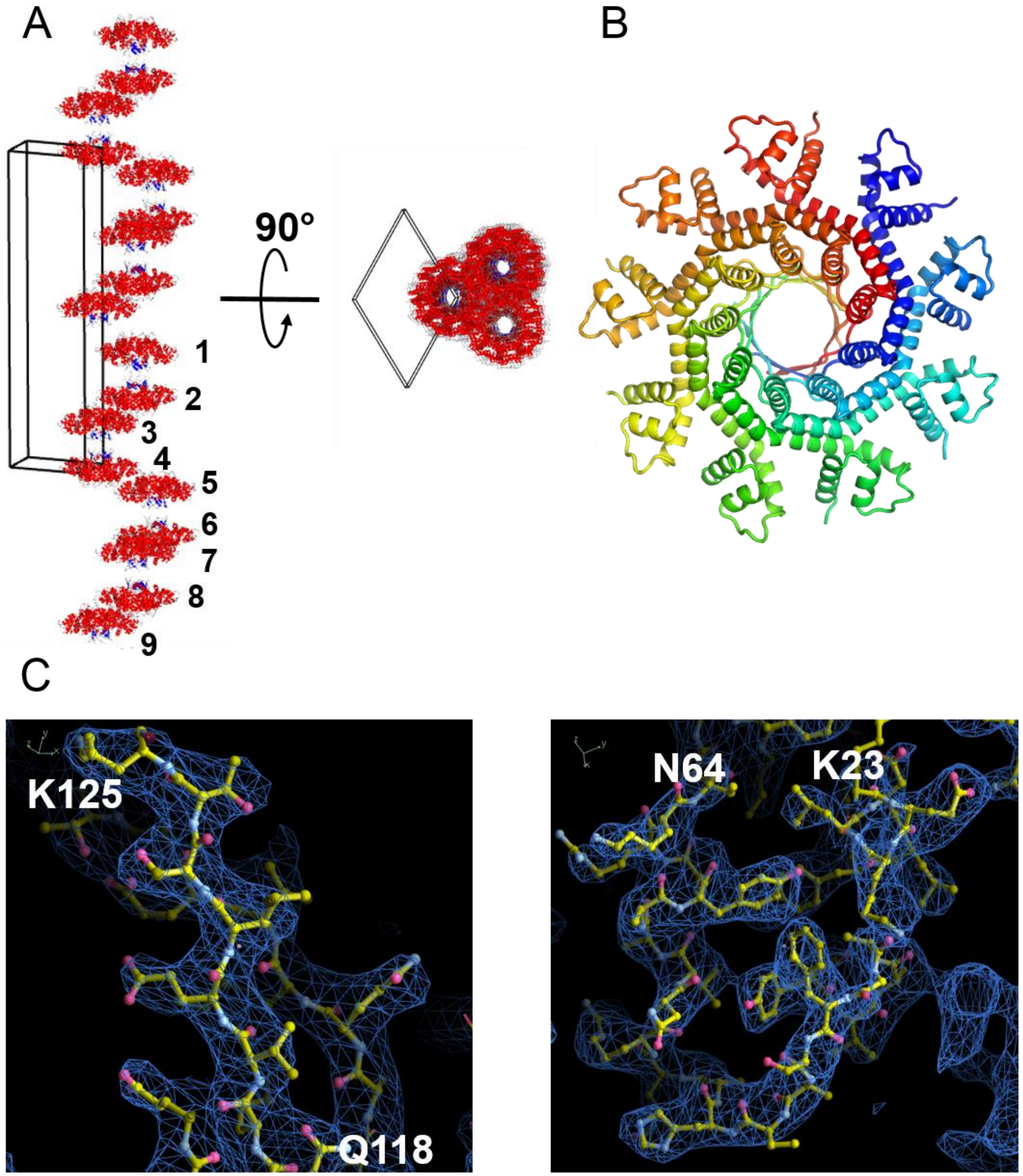

Supplementary Figure 5. Crystal structure of NV1 TerS. (**A**) Superhelical packing of NV1 TerS in the elongated P1 unit cell. The numbers 1-9 indicate the nine TerS copies in the triclinic unit cell. The likely point group of the data is P321 but due to twinning and pseudo-translation, the structure was solved and refined in P1. (**B**) Ribbon diagram of the NV1 TerS nonamer that was refined to a Rwork/Rfree of 25.9/29.1% at 3.95 Å resolution (**Table 2**). (**C**) Refined 2Fo-Fc electron density maps contoured at 1.5σ above background overlaid to the final model of NV1 TerS.

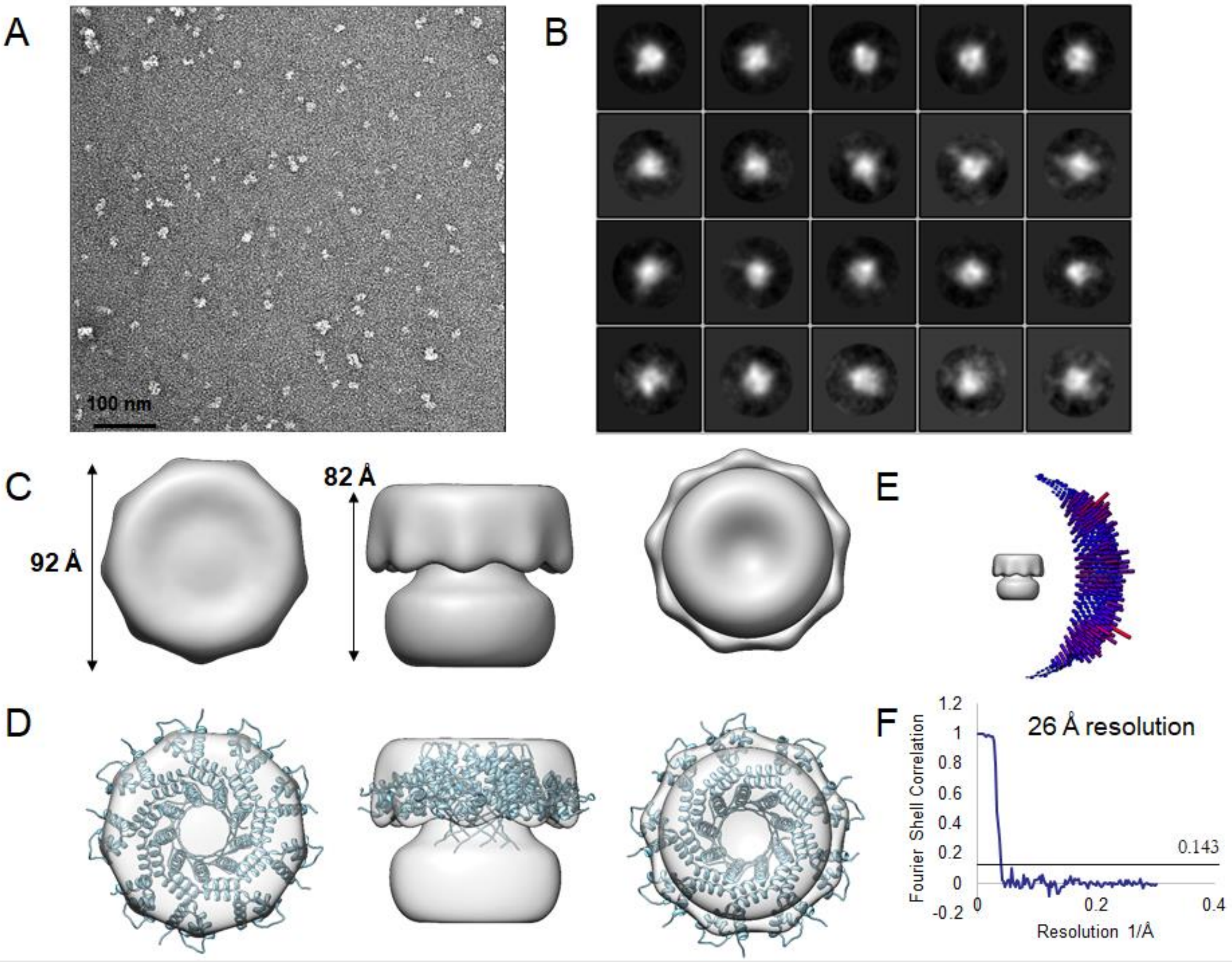

Supplementary Figure 6. Structural characterization of LUZ24 TerS by negative staining electron microscopy. (**A**) Electron micrograph of negatively stained LUZ24 TerS at 67,000 X magnification. (**B**) 2D-class averages of 3,000 particles processed with RELION-3.1 (4). (**C**) C9 symmetrized 3D density maps of LUZ24 TerS displayed in three orientations and (**D**) overlaid with the PaP3 TerS, represented as ribbons, fitted manually using Chimera (5). The extra density projecting at the C-terminus of TerS likely corresponds to the nine tails (residues 123-153) invisible in the crystal structure. (**E**) Angular distribution plot of all LUZ24 TerS particles that contributed to the final map. (**F**) FSC curve of unmasked LUZ24 TerS maps. Resolution at cut-off 0.143 = 26 Å.

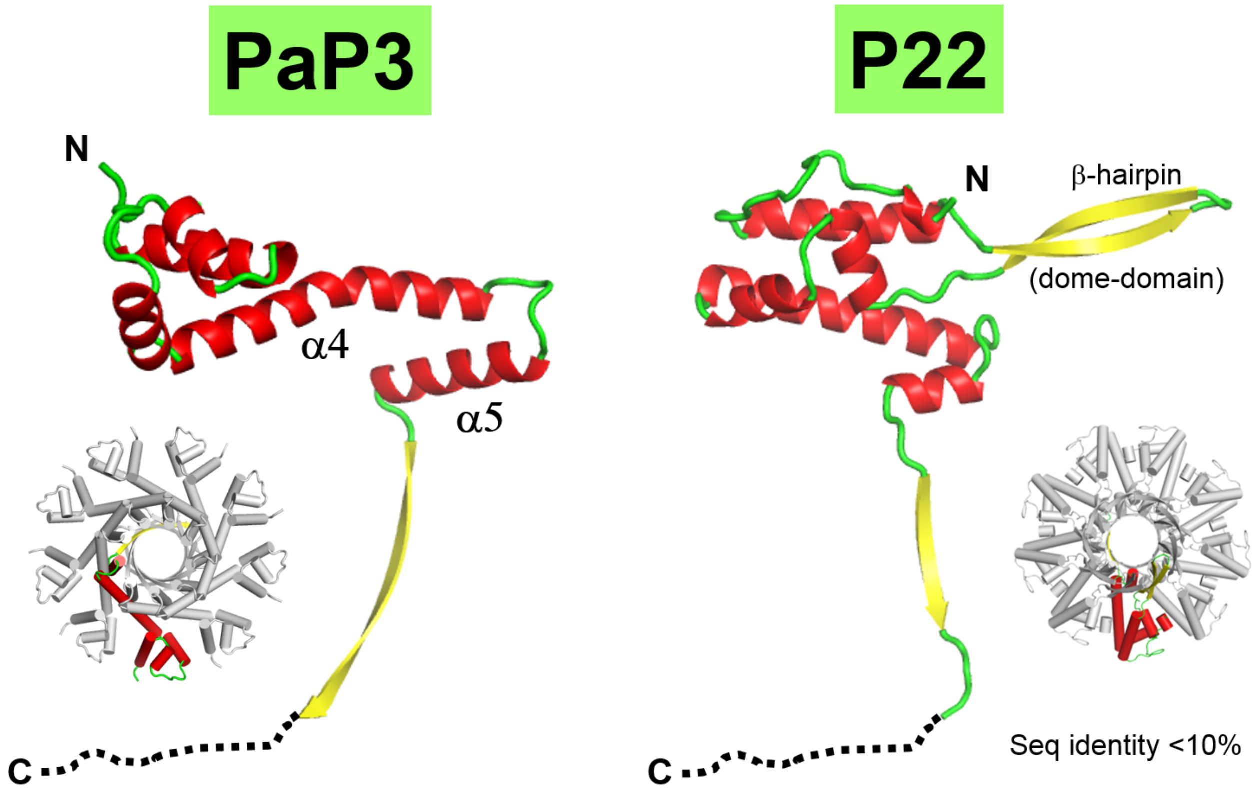

Supplementary Figure 7. Comparison of P22 versus PaP3 TerS. The solvent-accessibly surface buried upon assembly formation is smaller in PaP3 than P22 (29,980 Å^2 versus ~ 34,900 Å^2), underscoring a smaller protomer:protomer interface. Forty-three residues stabilize the interface between two protomers in PaP3 TerS versus 51 in P22, and both interfaces are dominated by hydrophobic contacts, as suggested by the negative solvation free energy gain upon the formation of the interface ($\Delta^i G$ ~22 kcal/M). The free energy of assembly dissociation (ΔG_{diss}) calculated by PISA (6) is smaller for PaP3 TerS (95.8 kcal/mol) than P22 TerS (167.2 kcal/mol).

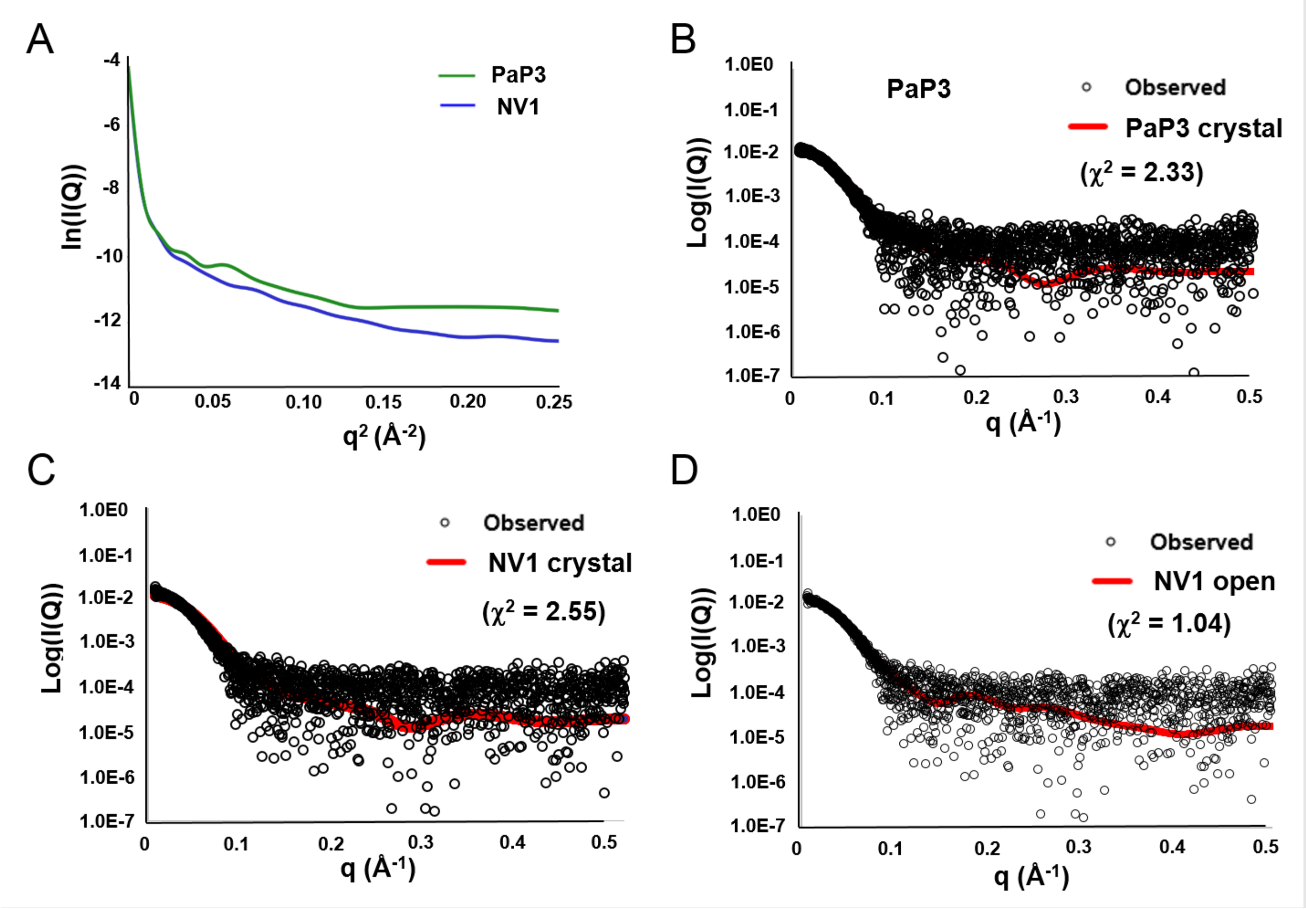

Supplementary Figure 8. PaP3 and NV1 SEC-SAXS analysis and agreement with crystallographic data. (**A**) Fitted Guinier plots of PaP3 and NV1 TerSs from **Fig. 4B** expanded to higher q values. Overlay of the experimental SEC-SAXS data (black circles) and scattering data (red line) calculated from the crystal structure of PaP3 TerS (**B**), NV1 TerS (**C**), and the open conformation of PaP3 TerS shown in **Fig. 5C** (**D**). The χ^2 values were calculated using the FoXS server (7).

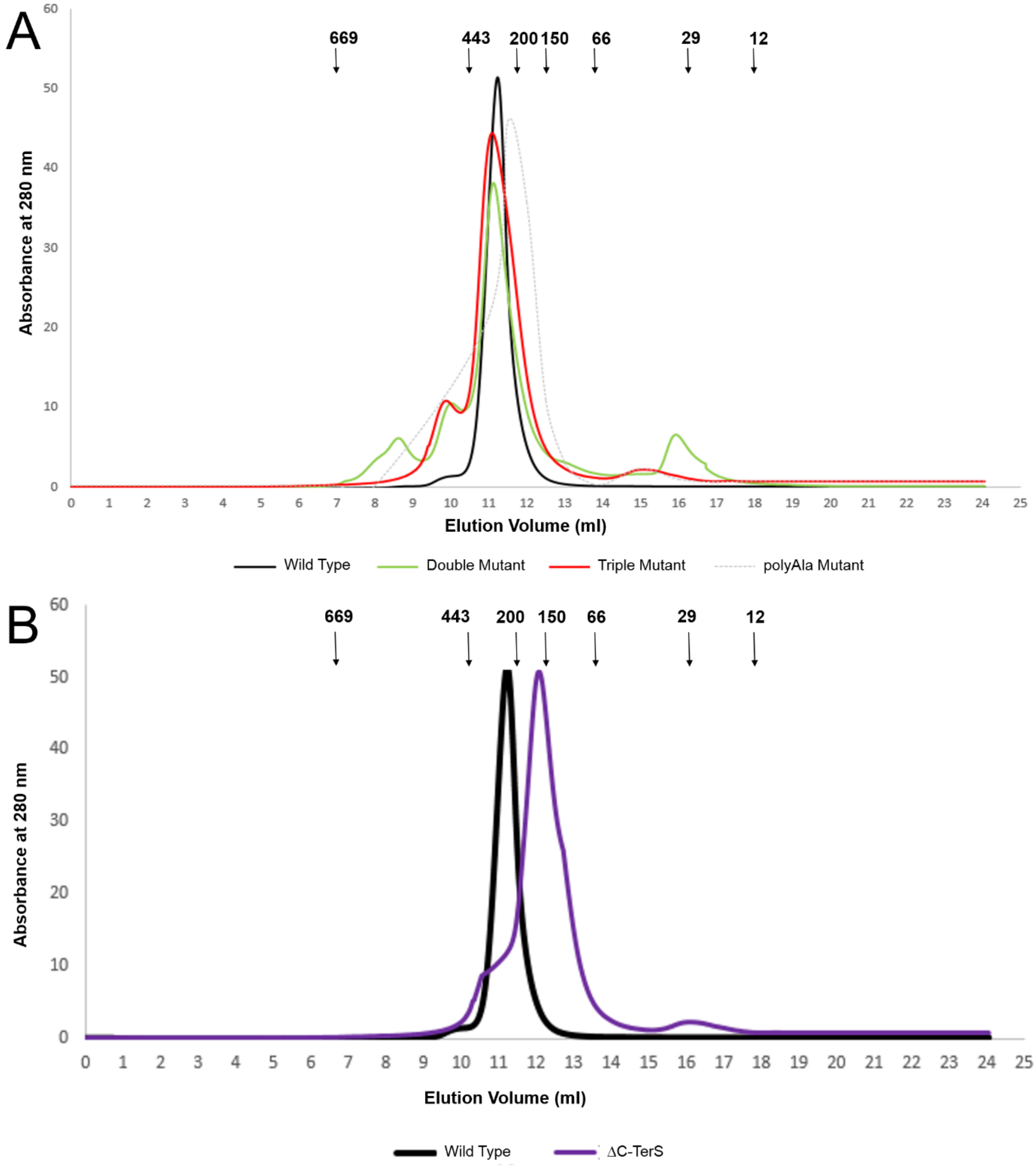

Supplementary Figure 9. SEC analysis of PaP3 TerS mutants in the DNA-binding domain (**A**) and C-terminal tail (**B**). Both Double Mutant (green) and Triple Mutant (red) migrated indistinguishably from the WT-TerS (black). In contrast, the polyAla mutant (dashed gray) was slightly shifted to the right, possibly consistent with a smaller oligomer. Likewise, ΔC-TerS was also slightly smaller, in agreement with the loss of ~ 9 x 31 = 279 residues. Arrows indicate the migration of molecular weight calibration markers.

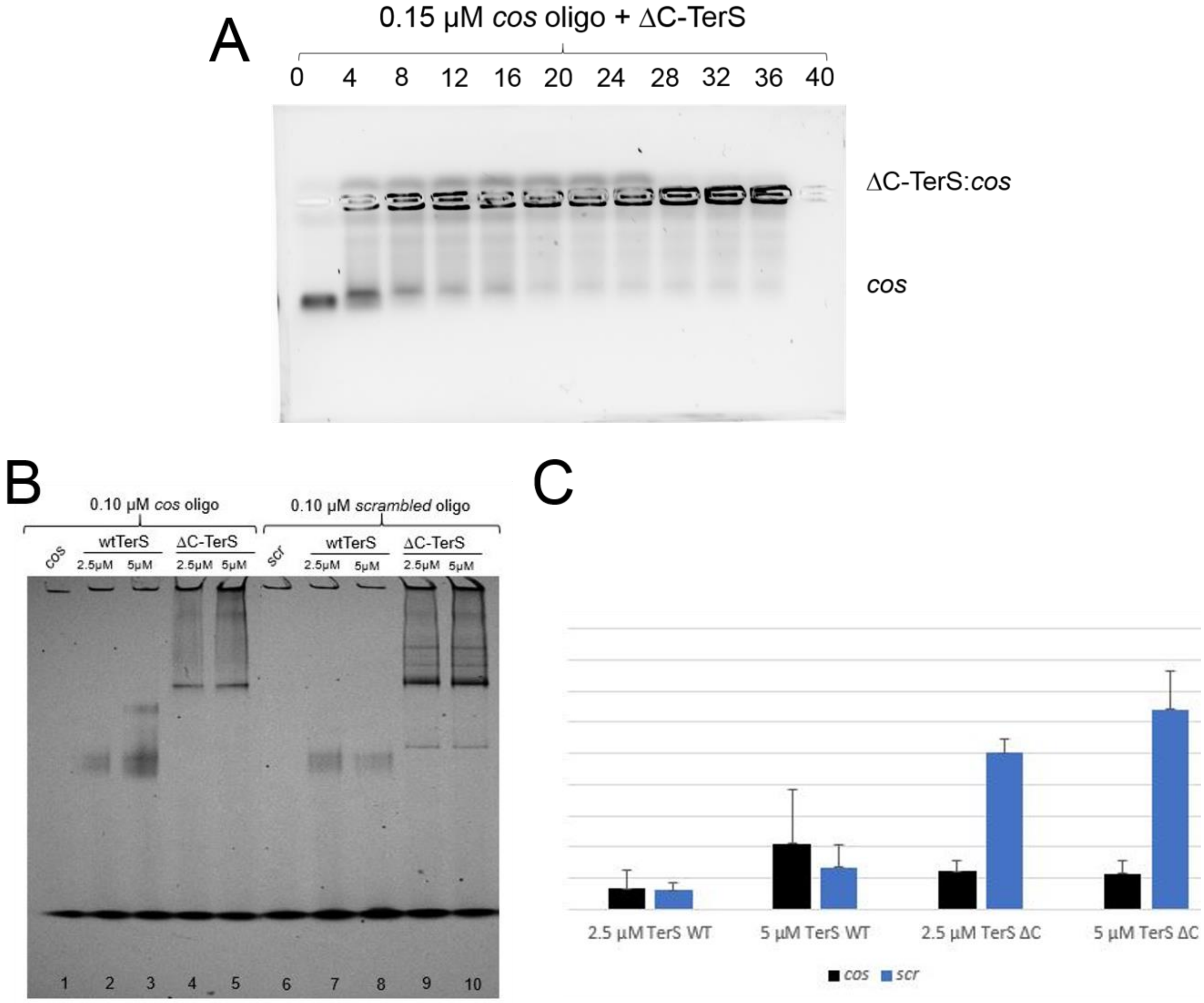

Supplementary Figure 10. EMSA of WT-TerS and ΔC-TerS. (**A**) DNA-binding assay on agarose gel as described for **Figure 6**. Increasing concentrations of ΔC-TerS were incubated with 0.15 uM *cos* DNA and analyzed on a 1% agarose gel. Unfortunately, ΔC-TerS did not migrate well on agarose and barely moved upwards, toward the cathode. Nonetheless, at 12 μM cos, almost all DNA was bound to the protein, suggesting ΔC-TerS retains full binding to DNA. (**B**) WT- and ΔC-TerS were incubated with *cos* or *scr* DNA at 37ºC and analyzed on a 4-16% acrylamide gel. The intensity of the bands that represent DNA-bound proteins was quantified and plotted in panel (**C**). Compared to WT-TerS, ΔC-TerS showed comparable binding to *cos* DNA and stronger binding to scrambled DNA.

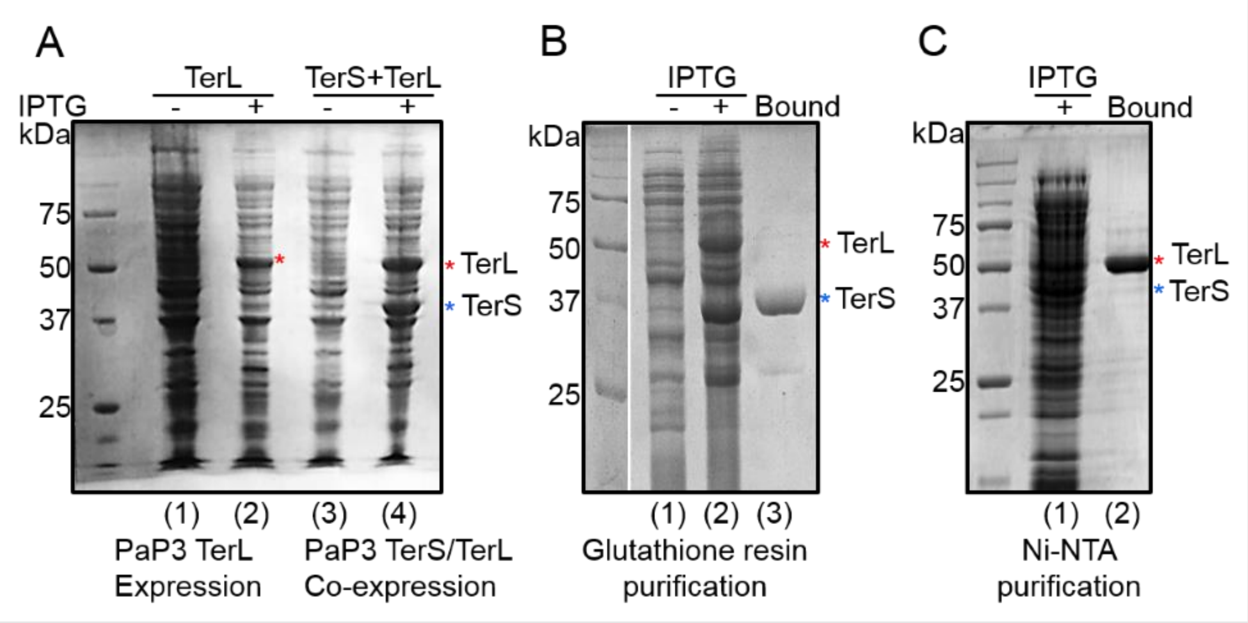

Supplementary Figure 11. PaP3 TerL and TerS do not stably associate when co-expressed in bacteria. (**A**) Expression of PaP3 his-TerL alone (lanes 1-2) and co-expression of his-TerL with GST-TerS (lanes 3-4) in E. coli LOBSTR cells. Purification of a bacterial lysate expressing his-TerL and GST-TerS over glutathione beads (**B**) and Ni-NTA resin (**C**) yielded just TerS and TerL, respectively, but failed to isolate a complex of the two proteins.

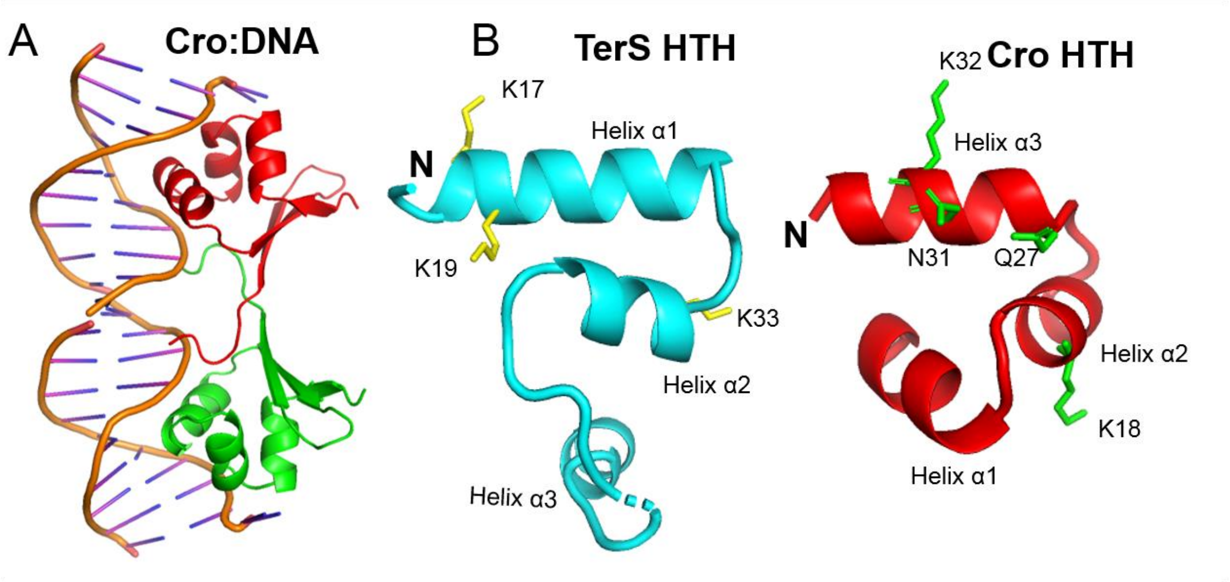

Supplementary Figure 12. Structural similarity between phage λ Cro repressor and PaP3 TerS HTHs. (**A**) Ribbon diagram of the heterodimeric phage λ Cro repressor bound to DNA (PDB 6CRO). (**B**) Structural comparison of PaP3 TerS HTH (cyan) and phage λ Cro (red). Colored in yellow (PaP3) and green (λ) are critical side chains involved in DNA binding.

REFERENCES

1. Przybylski, D. and Rost, B. (2002) Alignments grow, secondary structure prediction improves. *Proteins*, **46**, 197-205.
2. Robert, X. and Gouet, P. (2014) Deciphering key features in protein structures with the new ENDscript server. *Nucleic Acids Res*, **42**, W320-324.
3. Tong, L. and Rossmann, M.G. (1997) Rotation function calculations with GLRF program. *Methods Enzymol*, **276**, 594-611.
4. Zivanov, J., Nakane, T., Forsberg, B.O., Kimanius, D., Hagen, W.J., Lindahl, E. and Scheres, S.H. (2018) New tools for automated high-resolution cryo-EM structure determination in RELION-3. *Elife*, **7**. PMC6250425
5. Goddard, T.D., Huang, C.C., Meng, E.C., Pettersen, E.F., Couch, G.S., Morris, J.H. and Ferrin, T.E. (2018) UCSF ChimeraX: Meeting modern challenges in visualization and analysis. *Protein Sci*, **27**, 14-25. PMC5734306
6. Krissinel, E. and Henrick, K. (2007) Inference of macromolecular assemblies from crystalline state. *J Mol Biol*, **372**, 774-797.
7. Schneidman-Duhovny, D., Hammel, M. and Sali, A. (2010) FoXS: a web server for rapid computation and fitting of SAXS profiles. *Nucleic Acids Res*, **38**, W540-544.
